# Using QUASR-PCR as a field-based genotyping assay for a tick acaricide resistance marker

**DOI:** 10.1038/s41598-024-64401-0

**Published:** 2024-06-12

**Authors:** Christina Meiring, Michel Labuschagne

**Affiliations:** 1 Clinglobal, B03/04, The Tamarin Commercial Hub, Jacaranda Avenue, Tamarin, 90903 Mauritius; 2 Clinomics, Uitzich Road, Bainsvlei, Bloemfontein, 9338 South Africa; 3 Department of Microbiology and Biochemistry, Faculty: Natural and Agricultural Sciences, PO Box 339, Bloemfontein, 9300 Republic of South Africa

**Keywords:** Biological techniques, Genetic techniques, PCR-based techniques

## Abstract

A novel, turnkey, field-based workflow was developed and validated using *Rhipicephalus microplus* DNA as a template to detect the presence of the voltage-gated sodium channel *kdr* mutation. The field-based compatible workflow comprises manual sample homogenization for DNA extraction, PCR amplification of the targets in a closed tube, and end-point detection of the PCR products. An *R. microplus* species-specific assay was also included to confirm species identity and ensure the validity of the *kdr* mutation assay. The assays were sensitive and specific to the targets, and the workflow resulted in a turnaround time of approximately 1 h at a low cost. The novel combination of PCR with closed-tube and end-point fluorescent detection allows for easy conversion of existing conventional lab-based PCR assays into field-based detection assays. The incorporation of custom-designed 3D-printed components in the workflow provides easy adaptability and modification of the components for diverse nucleic acid detection workflows.

## Introduction

Most point-of-care (POC) tests (POCTs) for field-based applications are single-use serological tests that can be used to generate results in a short time frame (within 15 min) and are easy to perform. They are based on either antigen (testing current infection) or antibody (testing historical exposure to antigens) detection. The sensitivity of lateral flow device tests (LFDTs) for the detection of antigens is usually lower than that of nucleic acid-based amplification technologies ^1^. LFDTs are limited to antigen detection, which negates the discrimination power of genetic variation due to synonymous mutations and cannot easily discriminate between different isolates and single nucleotide polymorphisms (SNPs) present at the genetic level. Nucleic acid molecular detection assays can detect the presence of sample-derived foreign nucleic acids, host nucleic acids and genetic variations with high sensitivity and specificity. Nucleic acid amplification detection is commonly performed in well-equipped centralized laboratories utilizing high-throughput robotic handling of sample testing, making it unsuitable for POCT.

Several advancements have been made during the past decade to bridge the gap between nucleic acid amplification tests and their use in a POC environment, with polymerase chain reaction (PCR) and loop-mediated isothermal amplification (LAMP) technologies being the most advanced and applied technologies currently in use. PCR-based nucleic acid amplification tests are based on thermal cycling and normally require expensive and fragile amplification and detection units; however, portable instruments, such as the Biomeme Franklin and Tetracore 4/8, provide highly accurate results with limited hands-on time. The instruments are supported and used with their proprietary assay mixtures, and they employ hydrolysis probe-based chemistries for amplification detection. LAMP is a nucleic acid amplification technology and has been of interest due to its rapid reaction time (less than 1 h sample to results), sensitivity, specificity, low cost, and robustness, making it a competitive alternative to the gold standard PCR technique^2^.

The question is whether these new portable PCR-based technologies can be applied in the field, such as in parasite and insecticide resistance testing, which is an increasing problem in livestock and agriculture. The prevalence of tick acaricide resistance, especially in the host cattle tick *R. microplus*, is increasing worldwide, and is experienced in different geographies, including Africa. Resistance testing, according to FAO requirements, usually involves the highly specialized and time-consuming larval packet test (LPT) and the simpler larval immersion test (LIT) for phenotyping resistance testing^3^. The larval tarsal test (LTT) has emerged as a more efficient alternative test for a broader range of acaricides (compared to LIT) and is significantly quicker than the LPT assay but is still a reliable bioassay for detecting acaricide resistance^4,5^. The current study used a genotypic marker (associated with phenotypic resistance) as a predictor of acaricide resistance in ticks harboring the targeted genotype. As a proof of concept, we further developed a species-specific assay to prevent false negative results from incorrect species being targeted), as well as the *R. microplus* sodium channel *kdr* (C190A) SNP assay, using both LAMP- and PCR-based amplification technologies. We adapted this acaricide resistance diagnostic assay (ARDA) to be amenable to field application using a small-battery-powered mini16 thermal cycler and a custom-designed, 3D-printed fluorescence detection device and subjected it to field-based evaluation. An instructional video describing the complete workflow is available on YouTube under the search term “ARDA instructional video”.

## Results

### Genotypic target selection rationale and primer design

**Figure 1:**
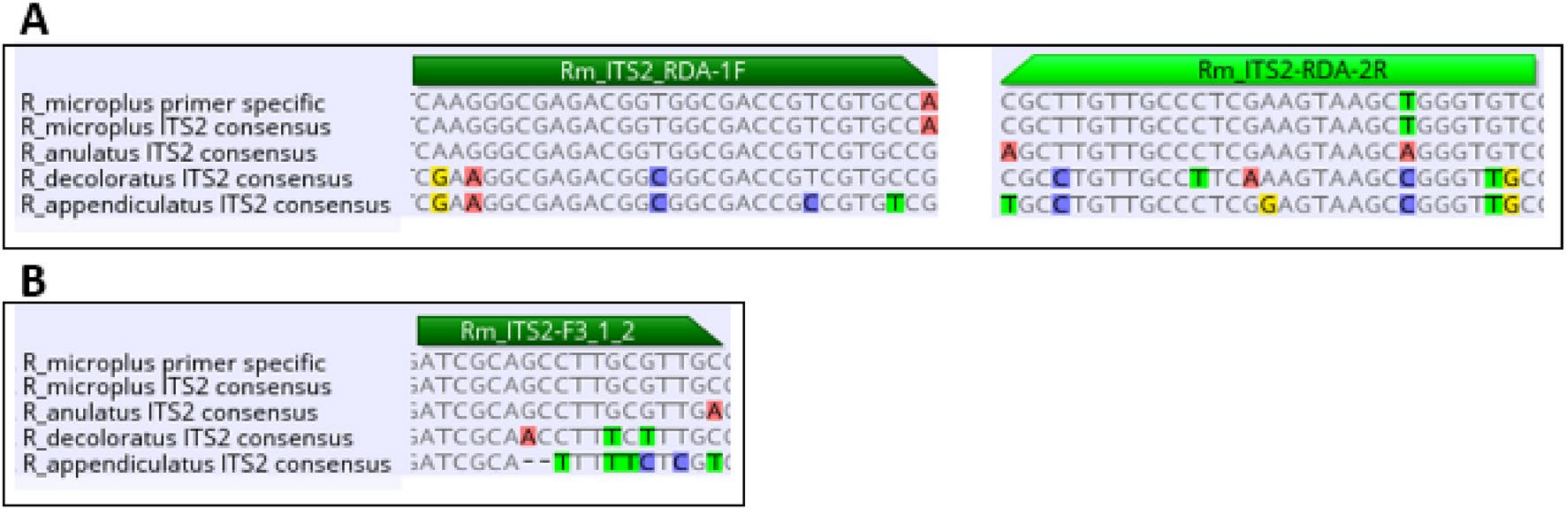
Allele-specific primers specific for the *R. microplus* ITS2 target region. PCR allele-specific primer binding sites indicating the 3′ mismatched oligonucleotides toward the nontarget regions (**A**). The *R. microplus* ITS2 specificity was based on the placement of the F3 primer (**B**).

Nucleic acid-based tests can be highly specific and reproducible and can easily discriminate between SNPs when appropriately designed. Morphological tick identification is a specialized field, and the morphologies of the engorged female cattle ticks *R. microplus*, *R. decoloratus,* and *R. annulatus* are morphologically indistinguishable by the untrained eye. Targeting of the *R. microplus* voltage-gated sodium channel *kdr* point mutation needed an *R. microplus-*specific assay to ensure that the correct species is being evaluated (limiting false negative results). It also served as a positive control to test whether the presence of species-specific amplifiable DNA was obtained from the sample. An abundance of genetic information is available in genetic repositories on *R. microplus*, but there is limited information for *R. decoloratus* and *R. annulatus,* with 150495, 458, and 744 GenBank nucleotide sequences available, respectively (15 March 2024). Mitochondrial DNA (mtDNA) is generally used for species identification due to its high copy number relative to the nuclear genome. Additionally, it can still be used in situations where DNA degradation has occurred due to suboptimal DNA preservation^6^. However, we decided not to target the mtDNA since the abundance of the mtDNA copies relative to the genomic target would not serve as an appropriate control for verification of amplifiable DNA derived from the nuclear genome. Tick nuclear rRNA ITS2 sequences were available for all three tick species of interest and were used in a multiple sequence alignment to identify sequence regions exclusively present in *R. microplus,* and primers were designed accordingly (Fig. 1). The species-specific PCR primer set was selected based on increased specificity toward the *R. microplus* ITS2 target and the relatively short length of the PCR target region (321 bp). This approach will result in shorter reaction times as well as increased ability to amplify fragmented DNA templates.

**Figure 2:**
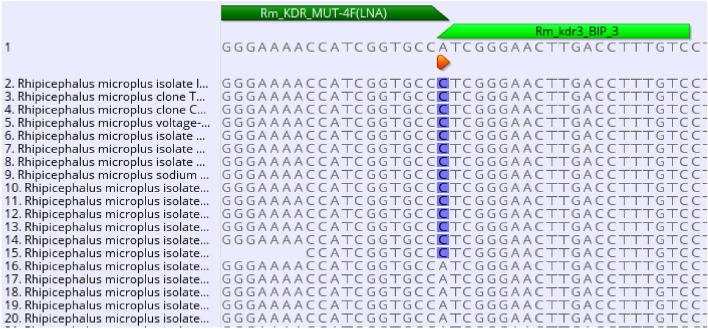
Allele-specific primers designed to be specific for the *R. microplus kdr* mutant C190A allele and primer binding sites indicating the 3′ mismatched oligonucleotides toward the nontarget regions [Rm_KDR_MUT-4F(LNA)] are indicated. The specificity of the *R. microplus kdr* LAMP was based on the placement of the BIP primer (Rm_kdr3_BIP_3), as illustrated in the figure, highlighting the *kdr* SNP binding region. The mismatched C nucleotide on the 3′-end of each oligo represents the wild-type (WT) allele, and the A at the same position represents the *kdr* mutant (MUT) allele.

The Rm_KDR_MUT-4F(LNA) primer was designed to be specific for the *kdr* mutation, and specificity was enhanced by the inclusion of a locked nucleic acid (LNA)-modified base^7^ as the penultimate 3’ nucleotide for the allele-specific forward oligonucleotide (Fig. 2). This *kdr* allele-specific forward primer was paired with a reverse primer (Rm_KDR_WT-1R) to amplify a 213 bp product.

### Conversion of PCR and LAMP assays to allow for end-point fluorescent detection

#### LAMP assay development and validation

The NEB WarmStart® Colorimetric LAMP 2X Master Mix was used for the development of the LAMP-based assays. This method afforded a simple colorimetric detection system visible to the naked eye with a room temperature setup, making it the ideal procedure for a field-based nucleic acid amplification assay. The primer design process for LAMP assays is more intricate than that for PCR and typically involves the use of design software to meet specific design criteria, ensuring that the desired efficiency is achieved. SNP discriminatory LAMP assays are notoriously challenging since different primers must match the sequence and distance criteria. Additionally, careful placement of the SNP-specific internal primer is crucial to encompass the SNP of interest, ensuring accurate allele discrimination. Several different primer combinations for the ITS2 and *kdr* targets were designed using PrimerExplorer V4. The primers were evaluated against the control templates for sensitivity and specificity. The final selected *R. microplus* ITS2 LAMP primer set yielded an *R. microplus*-specific assay result within 30 min of incubation at 65 °C (Fig. S1).

The *R. microplus* ITS2 colorimetric LAMP assay showed specificity toward the *R. microplus* template DNA within 30 min at 65 °C, and this specificity lasted up to 90 min. Nonspecific amplification was observed after 120 min at 65 °C (Fig. S1). The same discrimination against *R. annulatus* was observed for up to 90 min (data not shown). Agarose gel electrophoresis of the LAMP-amplified DNA exhibited concordance with the colorimetric observation and revealed the presence of nonspecific amplicons in the *R. appendiculatus, R. decoloratus* and no template control (NTC) reactions after extended incubation times (Fig. S2).

The *R. microplus kdr* assay was used to test linearized plasmid DNA and genomic DNA control samples for the presence of the *kdr* mutation. The assay exhibited specificity toward the *kdr* mutation in both the homozygous and heterozygous template DNA (Fig. S3). The heterozygous template resulted in a time-delayed color change, and an incubation time of 60 min was selected for reporting purposes. A consistent observation was the presence of a more orange product in the *kdr* genomic DNA samples compared to the yellow signal from the plasmid DNA controls. The orange color persisted from 30 to 60 min. No amplification was observed for the homozygous WT-only allele even at elevated copy numbers of 50,000 target copies per reaction (data not shown).

The limit of detection (LOD) was determined using linearized synthetic plasmids, and the LOD was calculated as 58 copies of the MUT allele per reaction (data not shown). LAMP reactions close to the LOD were subjected to agarose gel electrophoresis due to colorimetric inconsistencies (Fig. S4), which revealed the presence of a product without a clear color change, yielding false negative results.

Frequent opening (> 10 times) and use of the NEB WarmStart® Colorimetric LAMP 2X Master Mix also resulted in a noticeable fading of the pink color, which was particularly evident after the complete reaction mixture was prepared for template addition (Fig. S5). Furthermore, the reaction in tube 3 of the freshly opened LAMP master mix also resulted in a false positive result due to nonspecific amplification (agarose gel data not shown).

To address false positive and false negative colorimetric observations, as well as the fading of the indicator color in frequently opened samples, assay sensitivity and specificity were enhanced by incorporating fluorophores into the primers and introducing a complementary quencher to the fluorescently labelled primer^8^. The QUASR approach enhances fluorescence specificity because it ensures that fluorescence is generated only if the labelled primer is specifically incorporated into the product. Moreover, multiplexing can be simplified by employing two distinct fluorophores for the two target sequences (not achievable with the colorimetric assay).

The optimized QUASR fluorescence LAMP assay resulted in clear and specific detection of purified genomic DNA (10 ng) for the *R. microplus* ITS2 and *kdr* targets in separate reactions (Fig. S6). Multiplex detection yielded accurate results for ITS2 but resulted in false negatives and false positives for *kdr* detection. The sensitivity of the amplification using QUASR was also greater than that of the colorimetric assay. While no color change was observed in the ITS2 reactions, ROX-related fluorescence was detected in all the *R. microplus* isolates. The absence of a color change was because the multiplex assay generated a signal only at the elevated incubation temperature of 68.5 °C, leading to a reduced reaction efficiency, as indicated by the results observed in the single-target tube strips.

Several attempts using different reaction conditions and primer concentrations were undertaken to provide a reliable LAMP assay (colorimetric single-target detection or QUASR multiplex target detection) for the *R. microplus* ITS2 and *kdr* targets. No reproducible results were obtained, which led us to use single-target reactions.

Single-target LAMP assays for the ITS2 and *kdr* targets (which were optimized on purified *R. microplus* gDNA) were performed on crude DNA extracts from tick larvae, which also gave rise to false positive results when using a sequence-verified *R. microplus* isolate with no *kdr* mutation. The results indicated that the LAMP assays performed on the purified DNA accurately reported the genotypes. However, the *kdr* LAMP assay reported 2/4 false positives for the Rm_CVSA crude extract. Furthermore, no differences were detected between the colorimetric and QUASR signals in the false positive LAMP *kdr* assay signals obtained from the crude extract (Fig. S7).

Despite numerous optimizations and modifications, which were all limited to primer binding to a single locus, the nonreproducibility of the LAMP *R. microplus kdr* SNP assay using crude DNA as a template suggests that the assay is not fit for purpose as a field-based genotyping tool to predict acaricide resistance based on the *kdr* mutation as a function of synthetic pyrethroid resistance.

#### PCR assay development and validation

Historically, PCR has been the gold standard nucleic acid amplification technique for detecting specific DNA sequences in samples^9,10^. This method requires only a pair of primers and their binding sites, providing more freedom in the design and placement thereof within a sequence. The PCR process involves temperature cycle-dependent copying of the region flanked by the primers. Unfortunately, the application of PCR detection is limited to laboratory environments and requires bulky thermal cyclers with either very expensive and fragile optics or an additional gel-based analysis and capturing procedure, making the process time consuming and unsuitable as a practical field-based alternative to LAMP. Recent advances in PCR enzyme technology have led to the fusion of a double-strand binding domain with a DNA polymerase, resulting in a protein fusion that is significantly more processive and can tolerate common PCR inhibitors. One such enzyme, Platinum™ SuperFi II, combined with a proprietary buffer permits universal annealing and produces high yields of PCR products (www.thermofisher.com). This is directly applicable to our specific application. Due to its high processivity and inhibitor tolerance, it can complete a PCR run in less than 40 min and can accommodate the use of crude DNA extracts as templates.

**Figure 3:**
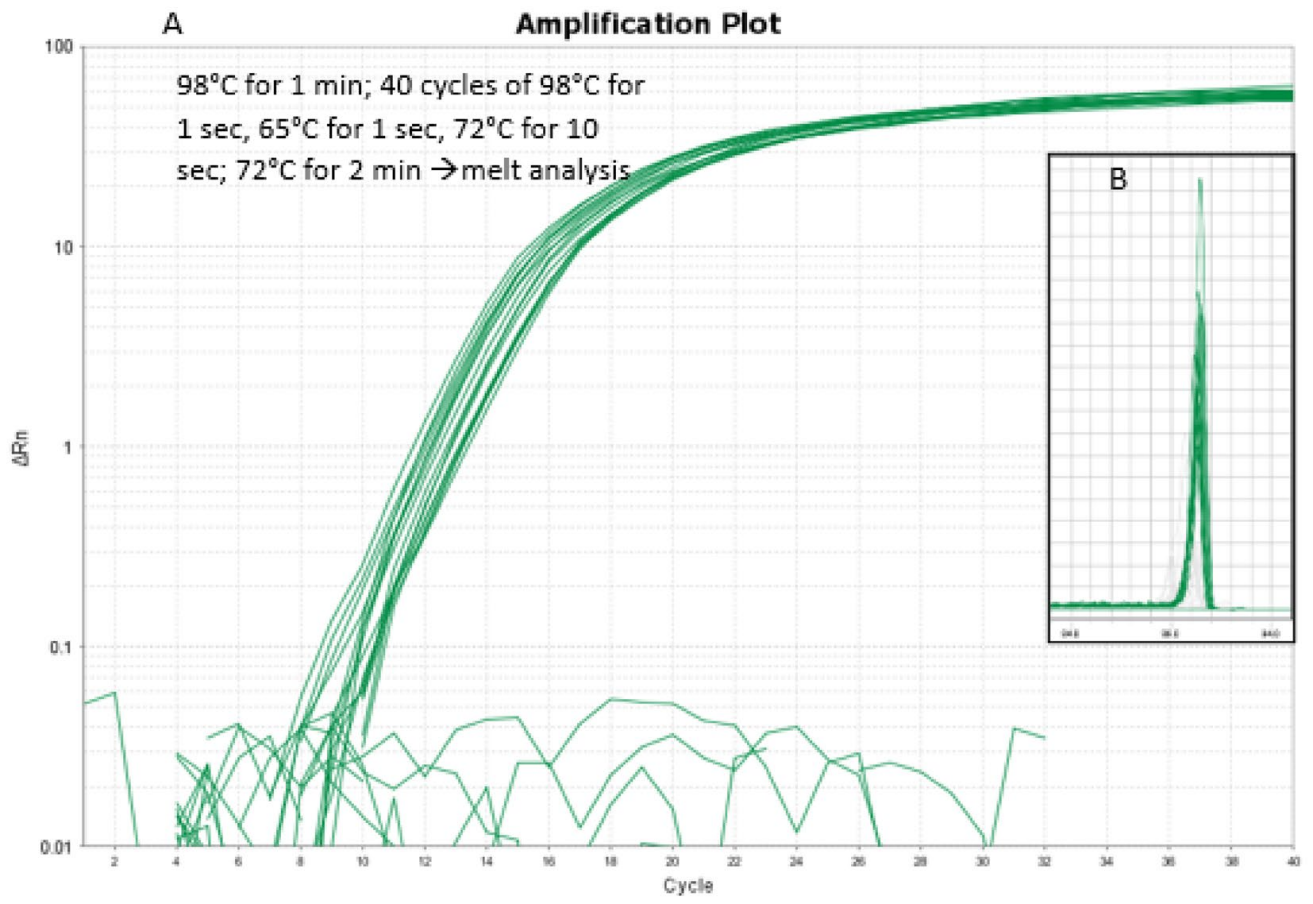
Real-time PCR amplification curve of tick DNA (**A**) using the Rm_ITS2_RDA_1F_2_(LNA) + Rm_ITS2_RDA_ 2R_2(LNA) primers with 3500 genome equivalents per reaction overlaid with specific thermal cycling parameters and the melt curve (**B**).

Primers specific for the *R. microplus* ITS2 region (Fig. 1A) were combined with Platinum™ SuperFi II PCR 2 × Master Mix and evaluated for specificity toward 10 ng of genomic DNA (3500 genome equivalents) using qPCR (Fig. 3). No off-target amplification was detected for *R. decoloratus, R. annulatus, R. appendiculatus, A. variegatum,* or *Bos taurus* template DNA after 40 cycles of amplification. The NTC reactions did not yield any detectable signal. The *R. microplus* DNA from different geographical origins was evaluated and yielded an average Ct value of 11.744 ± 0.52, indicating little variation in amplification efficiency between the isolates, and the melt curve analysis indicated a single melt peak for the *R. microplus*-amplified PCR products (Fig. 3).

**Figure 4:**
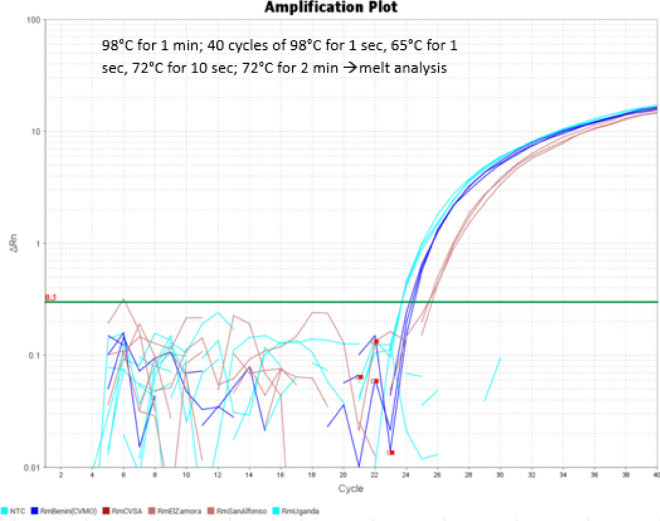
Real-time PCR amplification curve of tick DNA using primers Rm_kdr_MUT-4F(LNA) + Rm_kdr_WT-1R. No amplification was detected in the NTC, RmCVSA WT and San Alfonso WT isolates, but amplification was detected for the El Zamora, Benin, and Uganda *kdr* isolates, representing 1273, 2594, and 5203 genome copies per reaction, respectively.

Specificity testing of allele-specific primers for the *kdr* mutation in the *R. microplus* genome (Fig. 2) involved the examination of known plasmid DNA genotypes and *R. microplus* isolates with the *kdr* genotype using real-time PCR. The data indicated clear discrimination and allele-specific amplification of the *kdr* mutant sequence, with no detection of the WT sequence after 40 cycles (Fig. 4).

Crude DNA extracted from individual larvae was subjected to the *R. microplus* ITS2 assay as well as the *R. microplus kdr* allele-specific assay. Data generated using standard curve copy number calculations revealed that the ITS2 copy numbers were up to ten-fold greater than the copy number of the single-copy sodium channel gene (determined by PCR amplification of both the WT and MUT alleles with similar efficiencies) in crude extracts. The crude extracts contained more than 65,000 copies of each of the targets in a 1 µl template volume (Fig. S8). The average *R. microplus kdr* and ITS2 copy numbers, generated using a laboratory homogenizer and high-density zirconium oxide beads, were 259,219 ± 261,957 and 1,406,637 ± 1,276,028 copies/µl crude extract, respectively.

**Figure 5:**
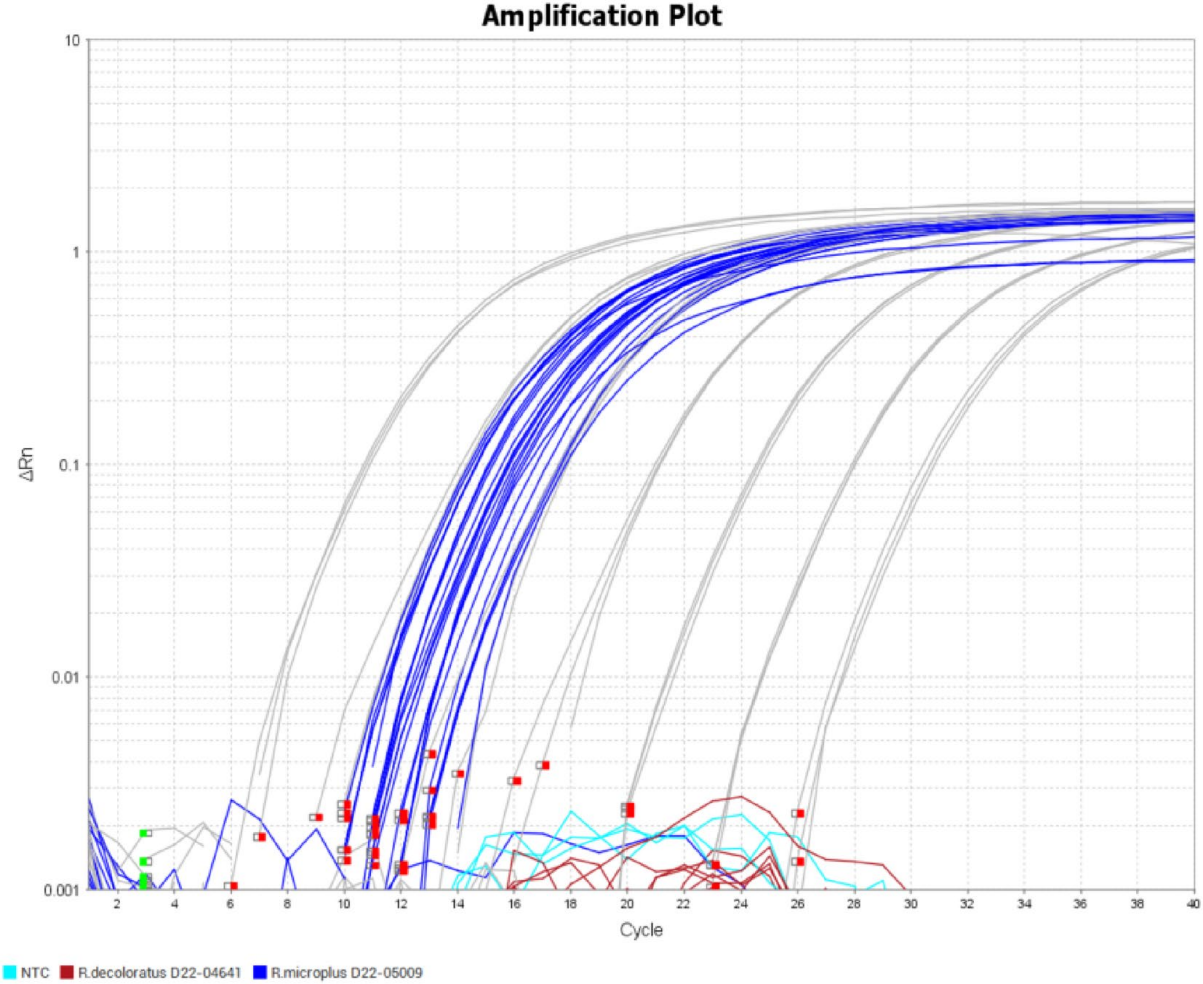
qPCR amplification curves of ITS2 target amplification from crude DNA extracts generated from individual larvae that were homogenized with zirconium oxide beads and lysed with proteinase K. The gray amplification curves represent the 7-point ten-fold diluted standard curve (covering a linear range of 40,000,000 copies down to 40 copies per reaction; R^2^ = 0.998, slope = − 3.348, efficiency = 98.9%).

The *R. microplus* ITS2 primer set produced no amplification signal with 1 µl of crude extract obtained from *R. decoloratus* individual larvae (Fig. 5), which is in concordance with results obtained from purified DNA (Fig. 3).

The PCR-based assays showed specificity toward the *R. microplus* ITS2 and *kdr* targets. In summary, the next step involves the conversion of these highly specific and robust assays into assay setups and formats that will be fit for field deployment.

### Field-based assay development and validation

#### DNA extraction from larvae

The proof-of-concept sample matrix chosen for DNA extraction was tick larvae, which were selected for compatibility with the LPT-derived biological samples available at the field test sites. For our assay, we needed to develop, optimize, and validate manual crude DNA extraction techniques suitable for field use. To access DNA for genotyping purposes, it is necessary to compromise the integrity of the larval exoskeleton. Laboratory-based sample crushing using a homogenizer and high-density beads was unsuitable for field-based assays. The extraction method must comply with the following parameters to minimize the use of instruments and reagents in the field-based DNA genotyping procedure:

##### Mechanical homogenization of tick larvae

Our field-based genotyping system employed a PCR approach, where the mini16 thermal cycler can accommodate 200 µl PCR tubes. This allowed for the use of standard 200 µl PCR tubes as the sole containers for DNA extraction. Physical disruption of the tick larvae exoskeleton was achieved by using a custom-designed 3D-printed white PLA pestle specially designed to fit within a 200 µl PCR tube (part of an 8-tube strip with individual caps). The individually capped strips ensure that single tube processing can be performed, limiting the risk of cross contamination between tubes. The mechanical homogenization efficiency was increased by including a small quantity of glass beads in the reaction mixture to serve as an abrasive component.

##### Enzymatic DNA release

Mechanically homogenized samples were subjected to enzymatic treatment with proteinase K in the presence of EDTA-containing buffer. This enzymatic treatment leads to the degradation of nucleases and the release of DNA from nucleosome complexes after the degradation of histones. The resulting thermal inactivation of proteinase K at 98 °C ensures that the inactivated enzymes do not degrade the DNA polymerase during the PCR process.

The white PLA used to print disposable pestles is manufactured from renewable raw materials and is a biodegradable polymer that is fit for purpose as a disposable component in the workflow. The white color provided better visual contrast to evaluate the degree of disruption of the individual larvae during homogenization. The DNA released from the larvae using the mechanical and enzymatic procedure indicated above resulted in adequate yields of the target template, as calculated by qPCR (Fig. S9). This is a robust and quick extraction method that may also have applications in various other field-based PCR detection procedures for resource-limited environments.

The results from the field-based homogenization method indicated that the amplifiable DNA extracted from individual larvae was comparable to that extracted via the laboratory-based technique. However, variations were evident among larvae from different isolates, and distinct differences were observed when different operators conducted field-based crude DNA preparation on larvae from the same isolate. The minimum *kdr* copy number detected was 83 copies/µl DNA extract and was within the limit of detection for the *kdr* assay after 40 cycles.

#### QUASR PCR

**Figure 6:**
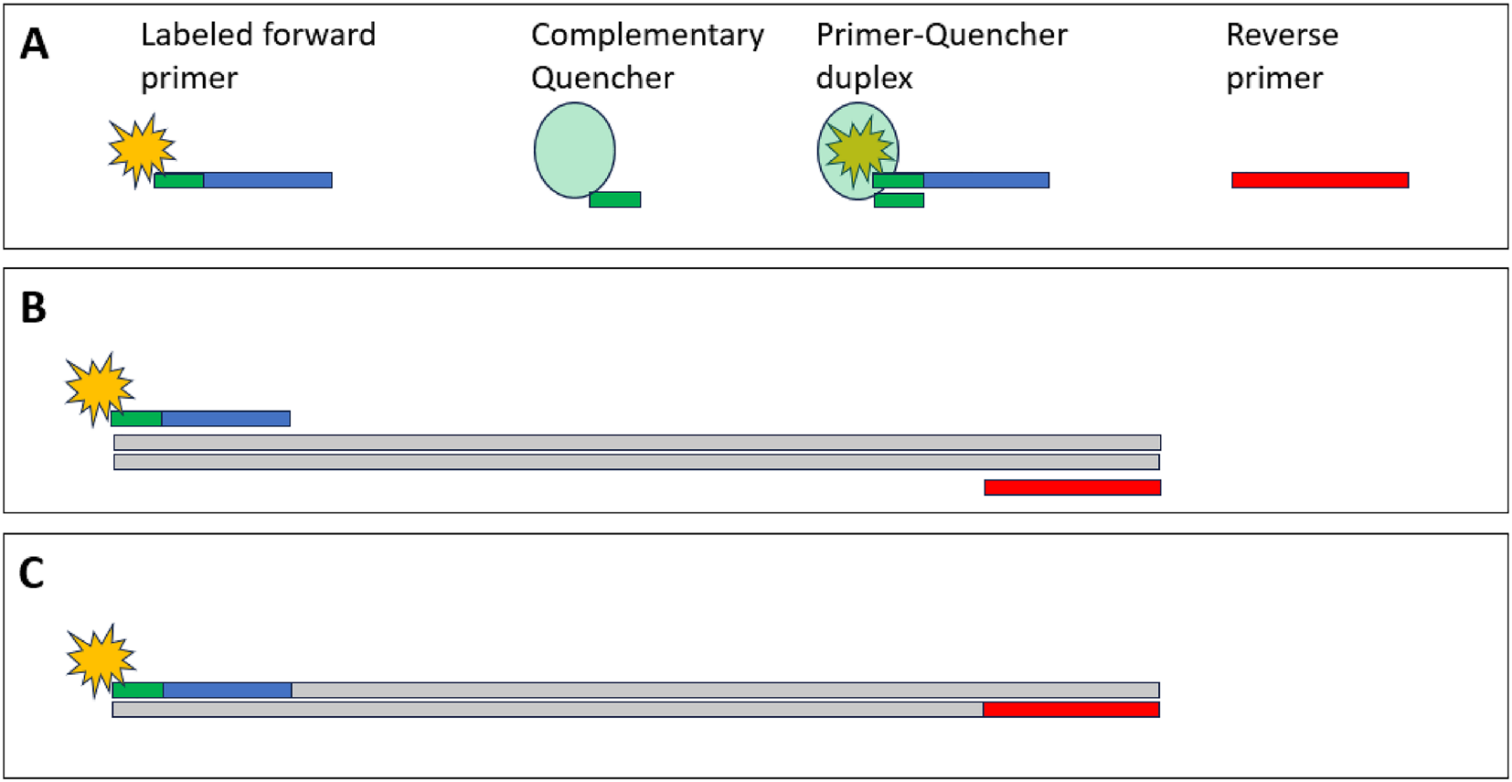
QUASR closed-tube detection method adapted for PCR. The primers and quencher needed for QUASR detection of PCR products: a fluorescently labelled forward primer, complementary quencher, labelled primer-quencher duplex, and standard reverse primer (**A**). The primer-quencher duplex dissociates during thermal cycling and allows the single-stranded labelled primer to hybridize with the target sequence to initiate PCR amplification of the target region (**B**). The fluorescently labelled primer is incorporated in the PCR product, and the double-stranded PCR product prevents hybridization of the quencher with the incorporated primer, resulting in fluorescence detection upon excitation (**C**).

The Quenching of Unincorporated Amplification Signal Reporters (QUASR) detection approach allows for single-step, closed-tube multiplexable detection of LAMP-amplified products^8^. Technical evaluation of the QUASR detection mechanism revealed that it could also be applied as an endpoint PCR product detection strategy with minimal modification to existing PCR primers. This entailed the addition of a fluorophore to the 5′-end of one of each of the PCR primers in a set, with a compatible 3′-end quencher (labelled, short oligonucleotide) complementary to the 5′-end of the fluorescently labelled primer. This will result in duplex formation at room temperature, allowing the quenching of fluorescent signals of unincorporated labelled PCR primers. No duplex formation will occur when the labelled PCR primer is incorporated in the double-stranded PCR product, resulting in the quencher not being near the fluorescent labelled primer to absorb the fluorescence when excited, resulting in fluorescence detection (Fig. 6). This end-point PCR detection represents a novel application of QUASR technology that, at the time of the QUASR PCR development, had not been previously described in the literature.

The QUASR *R. microplus* ITS2 and *kdr* allele-specific primer mixtures were used in standard qPCR assays (supplemented with ROX and Syto9) and compared to the normal nonfluorescent primers used during validation (section “PCR assay development and validation”). No differences were detected between the amplification signals obtained from the QUASR-based primer set and the original primer set for the *R. microplus* ITS2 and *kdr* targets. Closed-tube, end-point PCR products generated from the QUASR primer sets and the Platinum™ SuperFi II DNA polymerase master mix were excited with blue LED light, observed through an amber filter (DarkReader instrument) and compared to the qPCR amplification signals (Fig. S10). Bright yellow fluorescence from the incorporated FAM-labelled forward primer was observed in all individual larvae containing the *kdr* mutation.

The *R. microplus kdr* allele-specific assay was performed using a total of 24 selected individual *R. microplus* larvae (isolate D22-05009), and both the qPCR and QUASR assays were in concordance for all the larvae tested (data not shown).

The *R. microplus* ITS2-specific assay was assessed using a DNA extraction and testing protocol in a field assay to discriminate between DNA extracted from *R. microplus* and *R. decoloratus* individual larvae. A clear signal was observed in the PCR tubes containing crude DNA extracted from *R. microplus* individual larvae when compared to the background signal from the PCRs containing crude extracts from *R. decoloratus* individual larvae and the NTC reactions (Fig. S11).

#### Field-based QUASR PCR thermal cycling

Several thermal cycling devices that mainly use stable main electrical power are available on the market. The mini16 thermal cycling device from miniPCR is lightweight and portable and uses resistive heating and environmental fan-assisted cooling to perform thermal cycling. The unit can be powered by using main electricity or a portable 24 Ah rechargeable lithium battery. The combination of a mini16 and a battery pack weighs less than 1 kg (2.2 lb) and allows for easy and portable field-based operation. A fully charged battery will provide 3 h of mini16 cycling before it is recharged.

In field-based PCR amplification, 8-strip PCR tubes with individual caps are utilized to ensure the opening of a single cap at a time when crude lysate is added, limiting the risk of cross contamination. Each tube strip is prepared to contain an NTC in the second-to-last tube and a positive control in the last tube of the strip, which will serve as a reaction control to compare true positive fluorescent signals from potential background signals.

**Figure 7:**
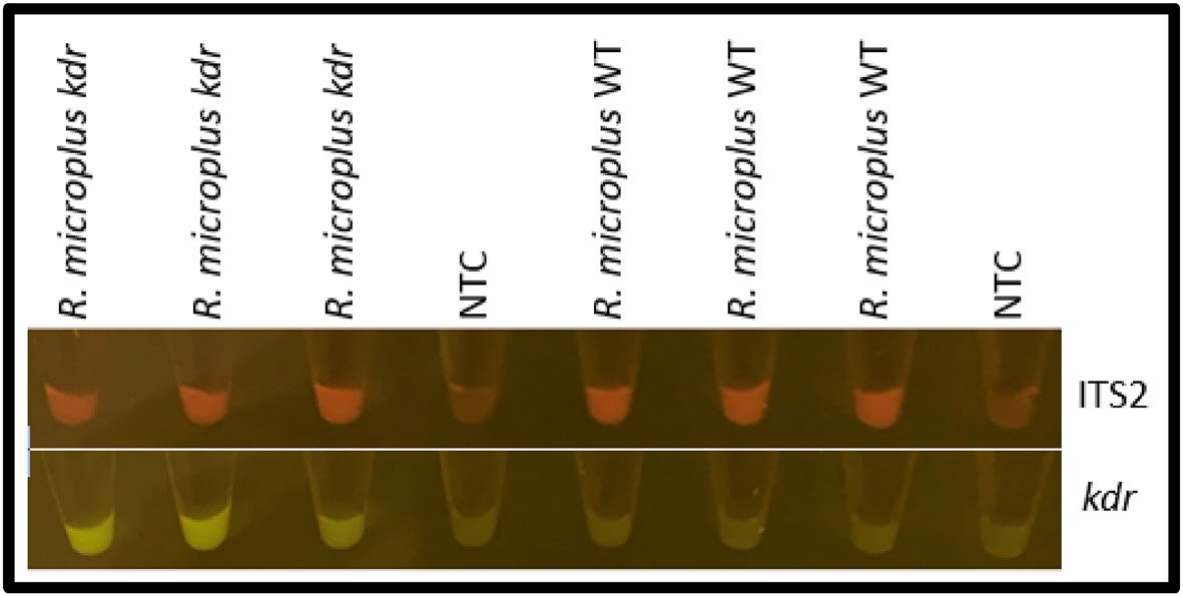
Mini16 thermal cycler amplification of *R. microplus*-specific ITS2 and *kdr* alleles using crude DNA extracts obtained from individual larvae, which included the optimized annealing temperature of 62 °C in the thermal cycling profile. Images were cropped to include the bottom of each tube containing the reaction mixtures.

The following thermal cycling profile was used with the mini16 thermal cycler: 98 °C for 1 min, followed by 40 cycles of 98 °C for 1 s, 62 °C for 5 s, 72 °C for 10 s and a final elongation at 72 °C for 2 min. This thermal cycling profile and reaction components were used to evaluate the amplification of the *R. microplus* ITS2 and *kdr* targets from crude DNA extracts from individual *R. microplus* larvae (Fig. 7). Both assays were able to accurately detect the genotypes of the individual larvae using an annealing temperature of 62 °C in the mini16 thermal cycler.

#### Field-based QUASR PCR detection

The optimized ARDA QUASR PCR conditions, implemented on the portable mini16 instrument for field-based amplification, required a field-based amplification detection device that would enable simple documentation and interpretation of the ARDA results.

**Figure 8:**
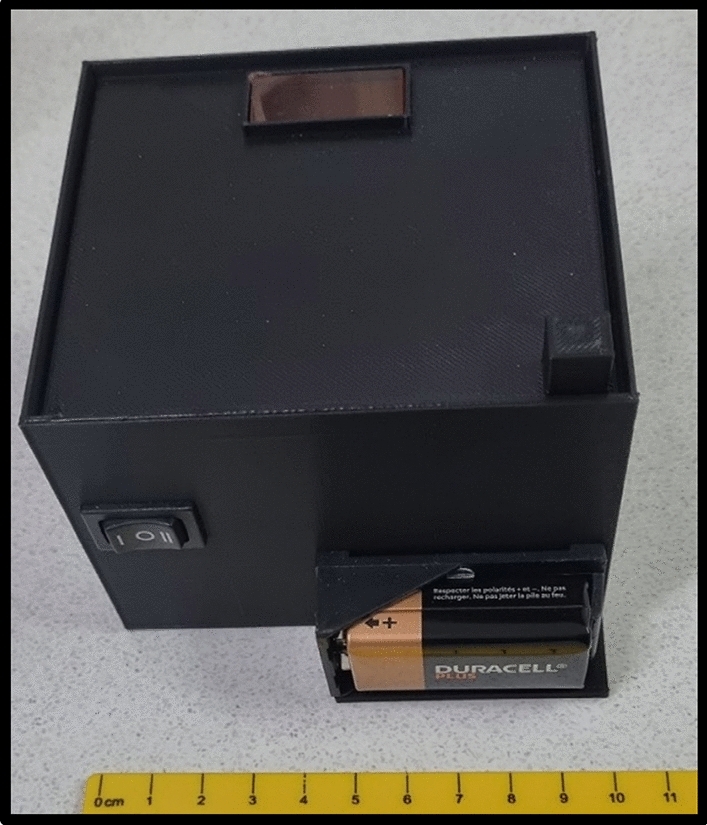
The field-based unit for the fluorescence detection of ARDA QUASR PCR.

A device weighing less than 200 g (including the 9 V battery) was designed, 3D printed, and optimized to allow for fluorescence detection of the ARDA QUASR PCR (Fig. 8).

The device consisted of a box-shaped external enclosure [10 cm (l) × 10 cm (w) × 8 cm (h)] containing an external 9 V secure battery attachment, an electrical power switch and 18 × 475 nm blue LEDs as the excitation source. Opposite to the LEDs, positioning pins for the tube strip holder (retainer) were integrated into the containment box. A removable lid structure equipped with an amber emission filter enabled the easy positioning of the tubes within the detection box. Once closed, a dark room environment is ensured, facilitating sensitive detection of fluorescence even under bright daylight conditions. The production cost of the complete detection unit (including labor) approximately USD 20.00.

Fluorescence detection and documentation are performed by placing the retainer with the tube strips onto the positioning pins in the box, switching the LEDs on and placing a camera (smartphone camera) over the amber filter and documenting the presence or absence of fluorescence from the reaction mixtures.

The master mixture of the final reaction mixture for *kdr* was prepared and aliquoted into 8-well PCR strips, followed by ARDA thermal cycling using a mini16 thermal cycler. The fluorescence was captured using a smartphone camera, and the PCR products were analysed using agarose gel electrophoresis (Fig. S12).

The documented fluorescence signals from the triplicate runs (Fig. S13) were analysed using ImageJ and normalized to the average fluorescence of all tubes and are expressed as a percentage of the normalized value (Fig. S13). The fluorescence analysis revealed that the observed and measured fluorescence intensities were lower in the tubes at the terminal positions of the PCR strips (positions 1 and 8), with the maximum fluorescence occurring in the middle of the strips (positions 4 and 5). This is most likely due to the positioning of the camera in the middle of the strip, creating an edge effect due to the longer distance from the middle to the terminal positions, since agarose gel analysis of the PCR product (except for run 2 position 8, Fig. S12) showed above average but comparable end-point PCR product yields for the tubes at the terminal positions. The difference in fluorescence is unlikely due to excitation luminescence overlap between the LED arrays since the 110° beam angle and the distance from the excitation source to the target results in complete overlap between the middle and side arrays. The addition of a diffuser screen could reduce the spotlight effect of the individual LEDs, but ImageJ calculated that the difference between the negative control and relatively weak fluorescence from limited product formation (a weak positive PCR) was more than 40% (data not shown). This provides enough discriminatory power to confidently call positive and negative PCRs.

LOD determination using the mini16 thermal cycler and the field-based detection unit resulted in a triplicate LOD of < 6 copies of *R. microplus* ITS2 and ≥ 40 copies of the *R. microplus kdr* allele (Fig. S14). The field-based ARDA LOD for the targets indicated the ability to reliably amplify field-based crude extracted DNA from larvae. The minimum target copy number was determined to be over 80 copies/µl of crude extract (Fig. S9).

**Figure 9:**
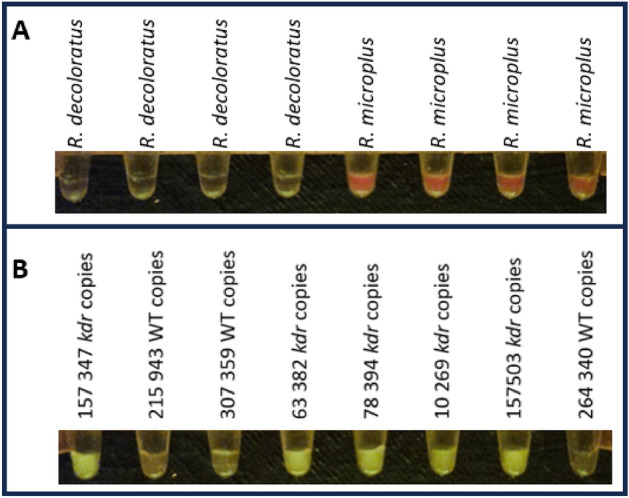
Field-based ARDA assays were performed with crude larval lysates using the mini16 thermal cycler and documented using the QUASR detection unit. The specificity toward the *R. microplus* crude DNA is indicated by the presence of red fluorescence (**A**), while the yellow fluorescence, indicative of the presence of the *R. microplus kdr* mutation, was in concordance with the qPCR data (**B**). Images were cropped to include the bottom of each tube containing the reaction mixtures.

The field-based ARDA assays were also evaluated using crude larval extracts from *R. microplus* and *R. decoloratus* and were shown to be in accordance with the qPCR and sequence-based genotyping results assigned to the analysed samples (Fig. 9).

DNA sequencing of the *kdr* locus from 23 individual larvae from two selected field isolates revealed full concordance between the *kdr* genotyping by sequencing and the presence of the ARDA PCR product for each individual larva.

#### Field-based ARDA results and user acceptance feedback

The field-based ARDA results were evaluated based on each site’s ability to perform functional PCR under the provided conditions (measured as the successful detection of a positive control signal from the control reactions) and the ability to extract amplifiable *R. microplus* DNA (measured as the successful amplification of the *R. microplus* ITS2-derived PCR product) (Fig. S16).

Analysis of data that met the inclusion criteria (successful amplifiable DNA extraction and valid control reactions) revealed user-observed and recorded false positive and false negative rates of 1.2% (2/167) and 2.4% (4/167), respectively. Analysis of the photographic documents indicated that some of the photos reported as false negatives were out of focus, leading to suboptimal data interpretation. This can easily be corrected by the addition of a set focus point in the camera image field within the detection box.

User acceptability results obtained from the three sites that evaluated the field-based detection kit were analysed using the data collected through Google Forms. The results indicated that the logistics, packaging, assay instructions and general operation of the field-based ARDA workflow were well received, but most of the sites struggled to operate the manual centrifugation device and found grinding of the individual larvae to be problematic. Interpretation of the results raised concerns due to some control failures that led to unsuccessful outcomes.

## Discussion

The increasing number of reports on *R. microplus* acaricide resistance, often involving resistance to multiple acaricide classes, emphasizes the need for simple and rapid strategies to determine resistance. These strategies are essential for managing acaricide use and for implementing tick management strategies. The larval packet test serves as the current gold standard test, but it is technically challenging and time consuming. The time period between sample collection and results can extend to several weeks, making it unsuitable as a rapid assessment test. The current work attempted to develop a proof-of-concept supplementary rapid acaricide prediction test. It is a DNA-based test targeting the known *kdr* SNP (C190A) in the *R. microplus para-*sodium channel that has been associated with synthetic pyrethroid resistance. Our recent work on phenotypic and genotypic characterization of *R. microplus* ticks showed and supported the importance of the *kdr* SNP on the increased resistance towards pyrethroids^11^ The *kdr* mutation was chosen as a proof of concept target due to its historic presence and association with synthetic pyrethroid resistance phenotypes observed in ticks and variety of insects with a wealth of data supporting this association^12,13^. Even though several other discovered sodium channel mutations have been associated with pyrethroid resistance, we believe that the single SNP discrimination assay developed in this study will provide the geographically diverse tick research community with the technical detail to adapt the target specific component to their target of interest. For the field-based application of the assay, it was essential for the procedure to be technically simple to perform and provide a rapid result in a turnkey package that can be used on site. LAMP and PCR-based SNP discrimination strategies were evaluated, and a PCR detection strategy (using a highly processive and inhibitor-tolerant DNA polymerase) was selected due to its reproducibility, accuracy, and increased possibility for multiplexing. Standard laboratory PCR was converted, using a novel approach, to allow for closed-tube fluorescent end-point detection without the need for capital-intensive and fragile instruments. The current assay employs (1) custom 3D-printed pestles for sample homogenization, (2) a portable battery-operated thermal cycler (mini16) that weighs less than 1 kg (2.2 lb) and is used for crude DNA extraction and PCR thermal cycling, and (3) a custom-designed 3D-printed detection unit weighing less than 200 g (0.44 lb). Additionally, a simple hand-operated centrifuge was also included in the workflow to ensure that the relatively small liquid volumes (≤ 20 µl) employed in the workflow could be easily and reliably collected at the bottom of the tube, optimizing the performance of the assay. The assay allows for rapid *R. microplus* species confirmation as well as the detection of the presence of the *kdr* mutation. All appropriate controls are included and serve as references for the validation of the results. A full instructional video of the turnkey package can be found using the search term “ARDA instructional video” on YouTube. The results obtained indicated a robust and reliable workflow and assay characteristics. The premixed lysis and QUASR PCR assays exhibited high sensitivity and specificity toward the targets of interest and were compatible with freezing and thawing followed by the detection of DNA from crude extracts. The current assay can also be used as a medium- to high-throughput laboratory-based assay without any modification, and detection can easily be performed in a conventional laboratory thermal cycler with a 96-well plate format and standard excitation and emission filters available in a molecular laboratory.

The current development provides (1) a simple and novel PCR detection combination of PCR with QUASR endpoint fluorescence detection that allows for easy conversion of existing laboratory PCR assays to field-based assays without significant optimization and (2) a custom-designed detection unit that allows for field-based fluorescence detection in broad daylight using a smartphone camera. The production cost of a complete detection unit was USD 20.25 (based on local cost using the exchange rate of 1 USD = ZAR 18.27 on 20 May 2024), and 3D printing files are publicly available. (3) Cost-effective field-based *R. microplus* species-specific and acaricide resistance prediction assays were designed. The total reagent and consumable costs for lysis and assays were USD 1.82 per sample analysed (comprising of USD 0.28 for extraction; USD 0.84 for the *R. microplus* specific assay; USD 0.71 for the *kdr* specific assay), and the results could be obtained within approximately 1 h of sample processing. (4) An end-point PCR detection workflow that provides a novel method to report PCR results and can be applied toward the field-based detection of genetic material from pathogens, parasites, and pests. (5) A novel molecular genotyping and detection approach that serves as a proof of concept is amenable to any known nucleic acid target. It provides the flexibility to perform multiplex reaction detection, further reducing the cost and time associated with running multiple samples. It also allows for medium- to high-throughput sample handling in a laboratory setup without any modifications.

Future efforts will entail multiplexing the different targets using QUASR-PCR as a simple, sensitive, specific, and cost-effective field-based molecular detection system.

## Methods

### Genotypic target identification and primer design

The *R. decoloratus* ITS2 sequence (MN266921) was used in a BLAST search to retrieve up to 968 similar sequences from GenBank (13 Aug 2021). The species-specific sequences for *R. microplus, R. annulatus, R. appendiculatus,* and *R. decoloratus* were extracted from the BLAST results and multiple aligned using the MAFFT algorithm^14^ to generate consensus sequence data that represent all the sequence variation in the alignments. The consensus sequences were subjected to a second round of multiple sequence alignment to serve as a template for *R. microplus*-specific primer design.

The *R. microplus para*-sodium channel *kdr* mutation, C190A^15^, was identified in the partial *R. microplus para*-sodium channel gene sequence (AF134216) and annotated, and the resulting partial sodium channel mRNA sequence was mapped to the *R. microplus* RmCVSA isolate genome assembly (accession number: JALIZG000000000) using Minimap 2.24^16^. The long read spliced alignment option was employed to map the mRNA sequence to the exons in the genome. A genomic region flanking the *kdr* locus by approximately 500 bp on each side was used as a template for PCR and LAMP primer design and used as a query during a BLAST search to assess the specificity of the primer binding site.

The primers used for the LAMP assay were designed using PrimerExplorer V4 software (http://primerexplorer.jp/elamp4.0.0/index.html). A sequence-specific F3 primer was manually positioned to be specific for *R. microplus* ITS2, and the sequence-specific BIP primer was specific for the *kdr* mutation. Three primer sets for each target were used (Table 1), and their specificity was evaluated. Different mismatches were introduced upstream of the 3’-end of the SNP in the target-specific primer in an attempt to increase genotyping specificity.

**Table 1:** Oligonucleotides used for LAMP and PCR detection of *R. microplus* ITS2 and *kdr* mutation targets.

Primer name	Sequence (5′-3′)	Supplier
Rm_7_E11-12-1F	TACATGTCCTGTCGAACAAGAGCCTTAC	Inqaba Biotec
Rm_7_E11-12-1R	CTTCTTCGCCTTCAATTGTTACAATG	Inqaba Biotec
Tick_uni_ITS2-1F	GTCGGCAACACGGACAGCACGCTGAAC	Inqaba Biotec
Tick_uni-ITS2-1R	GACCGACGGCGGACTACGACG	Inqaba Biotec
kdr_locus-Exon-4F	CTACGTGTGTTCAAGCTAGCCAAATCG	Inqaba Biotec
kdr_locus-Exon-2R	GTTTACTTTCTTCGTAGTTCTTGCC	Inqaba Biotec
Rm*kdr* LAMP		
Rm_kdr3_FIP	GCTTGAACACACGTAGCTGTCGACATGTCCTGTCGAACAAGA	IDT
Rm_kdr3_BIP_3	TAGCCAAATCGTGGCCTACCCTACAAAGGTCAAGTTCCCGCT	IDT
Rm_kdr3_F3	ACGTTACCGAGGCCAGAT	IDT
Rm_kdr3_B3	CACGGCGAAGATGAAGATGA	IDT
Rm_kdr3_LB(FAM)	FAM-TCTCTATCATGGGGAAAACCATCGG	IDT
Rm_kdr3_LB_rc_(IBQF)	CCCATGATAGAGA- IBQF	IDT
Rm_kdr3_LF	AAATACACACTGAGCGTAAGGC	IDT
RmITS2 LAMP		
Rm_ITS2_FIP_1	GGCAAAGCCTGGAGGAGGCCCCTCGAAGTAAGCTGGGT	IDT
Rm_ITS2_BIP_1	AGGGAGGTGAGGAGGCTGCGTGCTCTCCGCATCAACC	IDT
Rm_ITS2-F3_1_2	ATCGCAGCCTTGCGTAGC	IDT
Rm_ITS2_B3_1_2	CTTCGCGGCAAGTCTT	IDT
Rm_ITS2_LF_1(ROX)	ROX-TTCGAGCGCCCGGTCTA	IDT
Rm_ITS2_LB_1	TTTGCGGTTCGCTGCGTAC	IDT
Rm_ITS2_LF_1_Rc_1BHQ3	GGCGCTCGAA/3IAbRQSp/	IDT
RmITS2 QUASR PCR		
Rm_ITS2_RDA-1F_2(LNA)(ROX)	ROX-CAAGGGCGAGACGGTGGCGACCGTCGTGC+CA	IDT
Rm_ITS2_RDA-2R_2(LNA)	GACACCCAGCTTACTTCGAGGGCAACAAG+CG	IDT
Rm_ITS2_RDA-1F_2(LNA)3_RC_(BHQ2)	TTGCCACCGTCTCG**A**CCTTG-BHQ2	Inqaba Biotec
Rm*kdr* QUASR PCR		
Rm_KDR_MUT-4F(LNA)(FAM)	FAM-AGGGAAAACCATCGGTGC+CA	IDT
Rm_KDR_WT-1R	GTTGTGCGCTGAACGAATTTGAC	IDT
Rm_KDR_MUT-4F(LNA)_RC_1_1_(IBFQ)	ATGGTTTTCCCT- IBFQ	IDT

+Indicates the position of the locked nucleic acid (LNA) base immediately downstream; underlined nucleotides in bold indicate an introduced mismatch.

ITS2-specific PCR primers were designed for *R. microplus* and the *kdr* target by utilizing 3’ matches to the genome target, upstream mismatches, and modified bases to facilitate specificity toward the target templates (Table 1).

### Conversion of LAMP and PCR assays to allow for end-point fluorescent detection

LAMP and PCR assays were further modified using the Quenching of Unincorporated Amplification Signal Reporters (QUASR) approach^8^. Oligonucleotides were designed with different fluorophores and quenchers specific for each target amplicon for both LAMP- and PCR-based approaches (Table 1). Modifications and mismatches between the fluorescent primer and the quenching probe were incorporated based on experimental outcomes to ensure specific and sensitive fluorescence detection of the amplified target region.

#### Amplification reactions and optimization

All primers and labelled oligonucleotides used in the final reactions are listed in Table 1.

All LAMP reactions were performed in a final volume of 10 µl using NEB WarmStart® Colorimetric LAMP 2× Master Mix according to the manufacturer’s recommended reaction setup and conditions, with modifications where needed. LAMP products were also analysed using agarose gel electrophoresis to confirm visual observations. LAMP optimization entailed using different reaction temperatures, varying primer sequences and concentrations, and guanidine hydrochloride and dimethyl sulfoxide (DMSO) as additives. All photographic images were captured using a Samsung S23 smartphone camera with no editing of the captured photos.

Platinum™ SuperFi II PCR 2× Master Mix (Thermo Fisher Scientific) was used for all PCR-based assays according to the manufacturer’s recommendations, and the final reaction volume was 10 µl. Quantitative PCR (qPCR) evaluation and prescreening of primer sets were performed by supplementing 1.25 ml of Platinum™ SuperFi II PCR 2× Master Mix with 1 µl of 5 mM Syto™ 9 and 1.25 µl of 50 µM ROX (Thermo Fisher Scientific) to assess PCR product formation and melt curve analysis for quality control. Thermal cycling was performed according to the manufacturer’s recommendations, with FAM detection used during the extension thermal cycle and a melt curve analysis post-PCR from 60 to 95 °C at 0.015 °C/s increments on a QuantStudio™ 3 Real-Time PCR System (Thermo Fisher Scientific). PCR products were also analysed using agarose gel electrophoresis to confirm the qPCR results.

The *R. microplus*-specific ITS2 primer sets for both LAMP and PCR amplification were evaluated against genomic DNA from *R. microplus, R. decoloratus* and a synthetic control for *R. annulatus*. The *R. microplus* and *R. decoloratus* ITS2 regions were amplified from 10 ng of gDNA isolated from reference material provided by Clinvet South Africa (CVSA) using the Tick_uni_ITS2-1F and Tick_uni_ITS2-1R primers in a 10 µl final volume using Platinum™ SuperFi II PCR 2× Master Mix (Thermo Fisher Scientific). Amplicons were sequenced from both strands using Sanger sequencing, and molecular identification confirmed the identity of the reference material. The *R. annulatus* ITS2 region analogous to the *R. microplus*-specific ITS2 primer set assay target region was synthesized using oligonucleotide sequences generated by Primerize^17^ based on the GenBank accession MH464283. The phosphorylated synthetic PCR product was cloned and inserted into the Lucigen pSMART-HCKan vector (GenBank accession AF532107), and the sequence was confirmed using Sanger DNA sequencing. Plasmid DNA containing the *R. annulatus* ITS2 target region was linearized and diluted to 3500 copies using 10 ng/µl *R. appendiculatus* DNA to generate an artificial *R. annulatus* ITS2 template control sample. The *R. microplus*-specific ITS2 primer sets were evaluated using 10 ng of gDNA from *R. microplus* isolates from diverse geographic origins (Table 2).

**Table 2:** Tick DNA used for the *R. microplus*-specific ITS2 primer set evaluation.

Identifier	Species	Geographic origin	Source
AvCVMO	*A. variegatum*	Tanzania	Clinvet Morocco
Rannu	*R. annulatus*	Not applicable	Synthetic
RaCVSA	*R. appendiculatus*	South Africa	Clinvet South Africa
RdCVSA	*R. decoloratus*	South Africa	Clinvet South Africa
Rd1438	*R. decoloratus*	South Africa	MSD South Africa
RmCVSA	*R. microplus*	South Africa	Clinvet South Africa
RmLaMinita	*R. microplus*	Mexico	Clinvet Morocco
Rm961	*R. microplus*	Brazil	Clinomics, South Africa
Rm1425	*R. microplus*	Tanzania	Clinomics South Africa
Rm1426	*R. microplus*	Tanzania	Clinomics South Africa
Rm1427	*R. microplus*	Benin	Clinomics South Africa
Rm1429	*R. microplus*	Ghana	Clinomics South Africa
Rm1430	*R. microplus*	Nigeria	Clinomics South Africa
RmElzamora	*R. microplus*	Mexico	USDA

The *kdr* locus-containing region was amplified from 10 ng/µl *R. microplus* CVSA WT and 10 ng/µl *R. microplus* Benin MUT DNA using the Rm_7_E11-12-1F and Rm_7_E11-12-1R primers, subcloned and inserted into pSMART-HCKan and confirmed using Sanger sequencing. The plasmid DNA containing the *kdr* locus was linearized and diluted to 10 ng/µl, after which the WT and MUT samples were combined to generate a heterozygous WT/MUT standard. Dilutions were prepared from 10 ng/µl *R. appendiculatus* gDNA to evaluate specificity and sensitivity.

The kdr_locus-Exon-4F and kdr_locus-Exon-2R primers were barcoded and used to amplify DNA extracted from individual larvae using Platinum™ SuperFi II PCR 2× Master Mix. The barcoded PCR products were subjected to library preparation using the LSK109 kit and sequenced using an Oxford Nanopore Technologies Minion 9.4 flow cell. Nanopore signals (≥ q10) were base called using Guppy 5.0.11 with the super high accuracy model to generate sequence data, followed by demultiplexing and binning of the reads using miniBarcoder^18^. The binned reads were mapped against the reference sequence using Minimap2^16^. The individual genotypes were generated based on the number of *kdr*/WT reads originating from each individual.

The ability of the QUASR-incorporated PCR assay to amplify the *kdr* mutation from crude larval extracts was validated. Extracts were prepared by adding 2 × 2 mm diameter high density zirconium oxide beads to each well in a 96-well PCR plate containing 20 µl of lysis reagent [TE Buffer (10 mM Tris, 0.1 mM EDTA), pH 8.0; 0.8 units NEB proteinase K] per well. A single larva (from a population verified to be heterozygous for the *kdr* mutation) was added to each of the 94 wells. The last 2 wells served as controls. The larvae were homogenized by 2 × 30 s bursts of reciprocal movement with 4 strokes per second (stroke length: 28 mm), collected by centrifugation and subjected to lysis at 56 °C for 5 min and 98 °C for 5 min in a 96-well PerkinElmer 2720 thermal cycler. Crude lysate (1 µl) was added to 9 µl of QUASR PCR resulting in the following final composition: 1 × Platinum™ SuperFi II II PCR master mix, 625 nM labelled forward primer, 625 nM reverse primer and 1.875 µM quencher oligo. The reaction was cycled on a PerkinElmer 2720, fluorescence data were captured using a smartphone camera, and the PCR products were assessed using agarose gel electrophoresis. All photographic images were captured using a Samsung S23 smartphone camera with no editing of the captured photos. SYBR Safe containing agarose gels were placed on a Dark Reader blue transilluminator and the gel image captured through an amber filter using a Samsung S23 smartphone camera and the gel image was converted to grayscale.

### Field-based DNA extraction, amplification, and detection

Individual *R. microplus* larvae (used as biological starting material during this proof of concept) were placed in an individually capped PCR tube strip containing 20 µl of lysis reagent [TE Buffer (10 mM Tris, 0.1 mM EDTA), pH 8.0; 0.8 units NEB proteinase K] using a sterile 10 µl pipette tip prewetted in the lysis solution. Approximately 5 mg of 150–250-micron diameter glass beads (Blastrite Starbead® glass beads) was also included in the lysis solution to serve as an abrasive reagent during the grinding process. The larvae were homogenized by grinding using a custom 3D-printed disposable pestle (white PLA filament) until visual confirmation was achieved. The homogenized sample was placed in a mini16 thermal cycler (miniPCR) and incubated at 56 °C for 5 min and 98 °C for 5 min to allow for the release of DNA. PCR was performed using a final volume of 10 µl containing 1× Platinum™ SuperFi II PCR Master Mix and 1 µl of the crude DNA extract as a template.

miniPCR (www.minipcr.com) produces portable resistive heating and fan-assisted cooling thermal cyclers that are robust and simple to use. The mini16 (weighing 498 g) accommodates 2 strips of 8 × 0.2 ml PCR tubes and can be operated using a portable MiniPCR® Power Pack (weighing 500 g) allowing 3 h of thermal cycling on a single charge.

Tinkercad (Autodesk) was used for the design of the 3D printed components and accessories. The exported STL files were sliced in Ultimaker Cura (version 4.13.1) using a 0.2 mm layer height and 20% infill. The .gcode file was printed on a Creality Ender 3 3D printer using a black polylactic acid (PLA) filament for all components, except for the manual centrifuge body and disposable pestle where white PLA was used. Printing all components requires less than 130 g of filament and can be completed using this current setup in less than 20 h. All 3D-printed components described in this paper can be downloaded from thingiverse.com using the search term “ARDA 3D components”.

**Figure 10:**
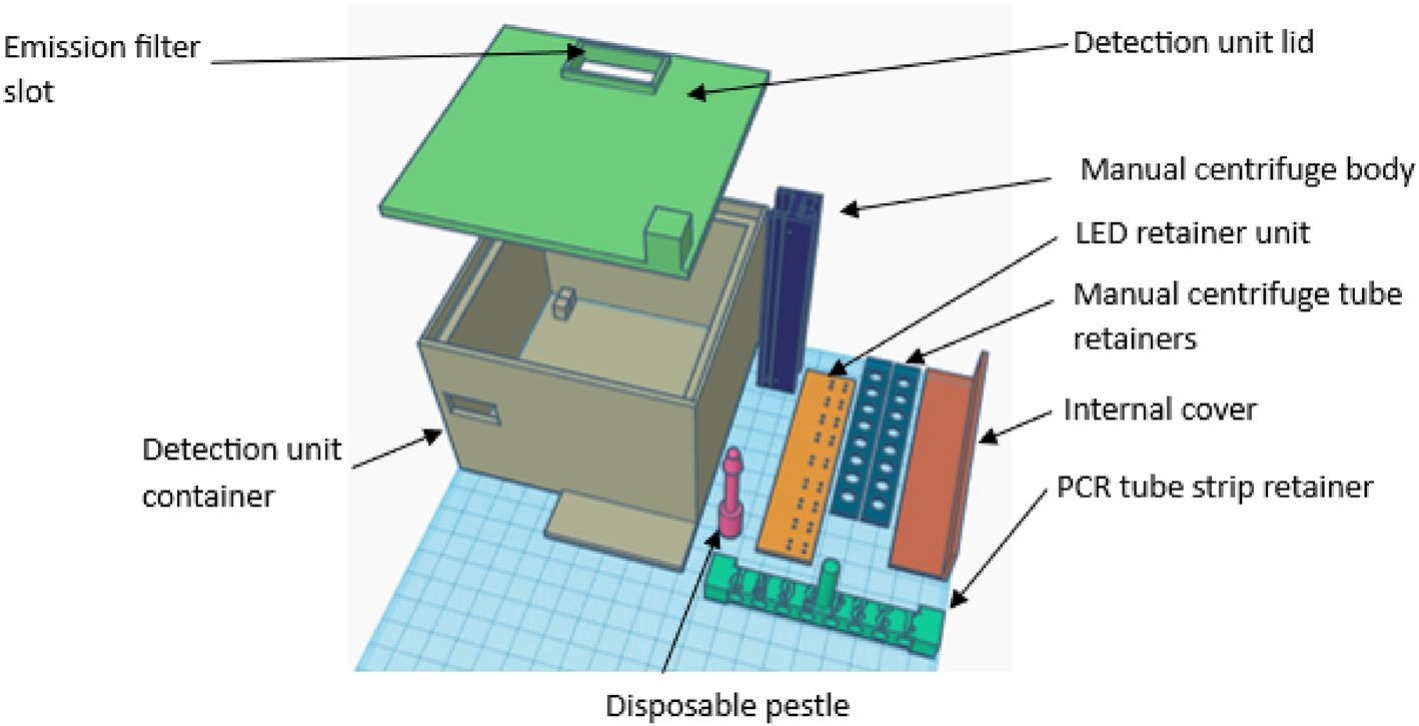
3D-printed components of the ARDA molecular detection unit and accessories needed to perform field-based nucleic acid detection assays.

The fluorescent signal detection unit was custom designed based on the fluorophores used in the final reactions (Fig. 10). The base unit and the lid were 3D printed using black PLA filament. The base unit included slots to accommodate a retainer containing the excitation source and a retainer (accordingly labelled to assist in sample tracking) for the correct positioning of the tube strip for fluorescence detection. A 9 V battery connection and retainer were added to provide electrical energy for the light emitting diode (LED) excitation source. The excitation source consisted of 18 blue LEDs (6 parallel connections of 3× LEDs in series; WW05E3SBQ4-W0; Wah Wang Holdings) with a peak wavelength of 475 nm and a 110° beam angle with a theoretical combined intensity of 22 lumens. An amber acrylic emission filter (Clare Chemical Research) located on the 3D-printed lid structure above the slot holding the reaction tube strip was used as the emission filter, and the filtered fluorescence was captured using a standard cell phone camera (Samsung S20, Samsung S22, Samsung A32, iPhone XR, OnePlus model 6). Images of fluorescent signals obtained from the detection unit were analysed using ImageJ^19^.

### Field-based evaluation and user acceptance feedback Field-based ARDA assay component preparation, shipping, evaluation, and user acceptance feedback

All reaction preparations were performed at Clinomics (Bloemfontein, South Africa) and were carried out using dedicated laboratories for PCR setup and DNA addition.

Lysis solution for crude DNA extraction was prepared in the PCR setup laboratory in a biological safety cabinet to minimize possible contamination. The lysis solution was prepared as a single batch and transferred into tubes 1–6 of an 8-tube PCR strip, each with an individual cap. The glass beads were added to tubes 1–6, the tubes were capped, and the tubes were stored at − 20 °C for future use.

The field-based use of a nucleic acid detection test requires control reactions to ensure that the obtained results are valid. The minimum requirement is an NTC and a PC to be included in each amplification run. The ARDA NTC will serve as a fluorescence background control for use as a reference, whereas the PC will serve as the reference fluorescent level expected from a positive sample when the reaction conditions are met. A PC’s failure to fluoresce above the background level of the NTC will invalidate the results from the specific run. The DNA-free reaction mixtures were prepared in the dedicated PCR setup laboratory to ensure consistency and limit the chances of contamination. The reaction mixtures were aliquoted in 9 µl volumes into tubes 1–7 of an 8-tube PCR strip. TE buffer (1 µl) was added to tube 7 to complete the NTC. After the individual PCR tube caps were closed, the tube strips were transferred to a dedicated DNA addition laboratory, where a single batch premix of positive control template and reaction mixture was added to tube 8. The tubes were capped and stored at − 20 °C for future use.

Frozen reagent (lysis solution and QUASR PCR premixes) tube strips were thawed and evaluated on individual larvae for amplification of the ARDA targets using the provided protocol (Fig. S15). Both assays were performed as expected and can be performed under cold chain conditions for field applications.Field-based detection reagents and instruments (section “Field-based DNA extraction, amplification, and detection”) were tested for shipping stability at 25 ± 4 °C for 7 days and were then functionally tested. A turnkey ARDA test kit was shipped to three sites within South Africa and provided with written and video instructions. The sites were then required to perform the ARDA. DNA was extracted from 24 larvae of each of the three LPT-characterized isolates, followed by PCR detection of the extracted DNA. The sites captured all the data, and the results were interpreted according to the provided instructions. All QUASR-PCR tube strips were shipped back to Clinomics for agarose gel electrophoresis quality control of each reaction and compared to the results captured by the three sites. The data inclusion criteria were based on successful amplifiable DNA extraction (based on the *R. microplus* ITS2 PCR product signal) and successful control reactions based on both *R. microplus* ITS2 and *R. microplus kdr* PCR product signals.

A Google Forms user acceptance survey was also conducted based on communication, shipping, packaging, and assay execution.

### Supplementary Information

Supplementary Figures.

## Data Availability

All data generated or analysed during this study are included in this published article and its supplementary information files. Any additional information requests can be sent to ML.
