# Publisher Correction: Using QUASR‑PCR as a field‑based genotyping assay for a tick acaricide resistance marker

**DOI:** 10.1038/s41598-024-67082-x

**Published:** 2024-07-11

**Authors:** Christina Meiring, Michel Labuschagne

**Affiliations:** 1 Clinglobal, B03/04, The Tamarin Commercial Hub, Jacaranda Avenue, Tamarin, 90903 Mauritius; 2 Clinomics, Uitzich Road, Bainsvlei, Bloemfontein, 9338 South Africa; 3 Department of Microbiology and Biochemistry, Faculty: Natural and Agricultural Sciences, PO Box 339, Bloemfontein, 9300 Republic of South Africa

Correction to: *Scientific Reports* 10.1038/s41598-024-64401-0, published online 12 June 2024

In the original version of this Article, Affiliations 1 and 2 were incomplete.

Affiliation 1: ‘B03/04, The Tamarin Commercial Hub, Jacaranda Avenue, Tamarin 90903, Mauritius.’

now reads

Affiliation 1: ‘Clinglobal, B03/04, The Tamarin Commercial Hub, Jacaranda Avenue, Tamarin, 90903, Mauritius.’

And

Affiliation 2: ‘Uitzich Road, Bainsvlei, Bloemfontein, 9338, South Africa.’

now reads

Affiliation 2: ‘Clinomics, Uitzich Road, Bainsvlei, Bloemfontein, 9338, South Africa.’

The original Article has been corrected.

