# Supplementary material for: Using QUASR-PCR as a field-based genotyping assay for a tick acaricide resistance marker
Source: Sci Rep. 2024 Jun 12;14:13584. doi: 10.1038/s41598-024-64401-0 (PMC11169234; doi:10.1038/s41598-024-64401-0)
Supplement: Supplementary file 1 — Supplementary Figures. [file 41598_2024_64401_MOESM1_ESM.pdf]

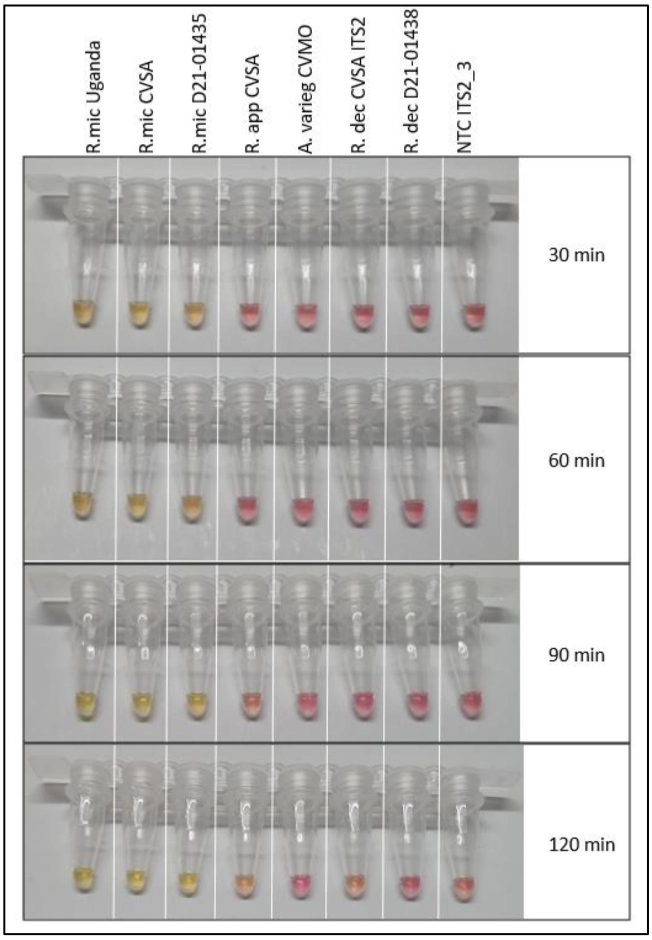

Figure S1. Colorimetric LAMP assessment of the final *R. microplus* ITS2 assay developed for the detection of *R. microplus*-derived genomic DNA. Each reaction contained 10 ng genomic DNA equal to approximately 3750 genome equivalents (using a genome size of 2.4685 Gb) and was incubated for 2 hours at 65°C and photographed every 30 min.

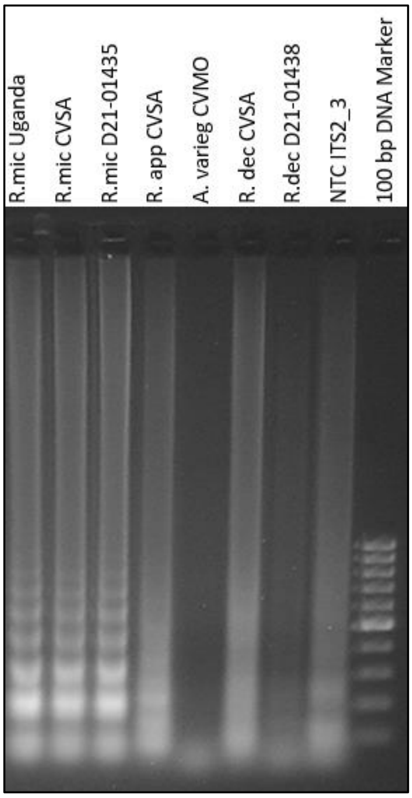

Figure S2. Agarose gel electrophoretic separation of the *R. microplus* ITS2 LAMP products after two hours of incubation at 65°C. The typical specific concatenated LAMP amplified products can be observed for the *R. microplus* template DNA (lanes 1 – 3), where nonspecific smears were observed for *R. appendiculatus* CVSA (lane 4), *R. decoloratus* CVSA (lane 6) and the NTC (lane 8).

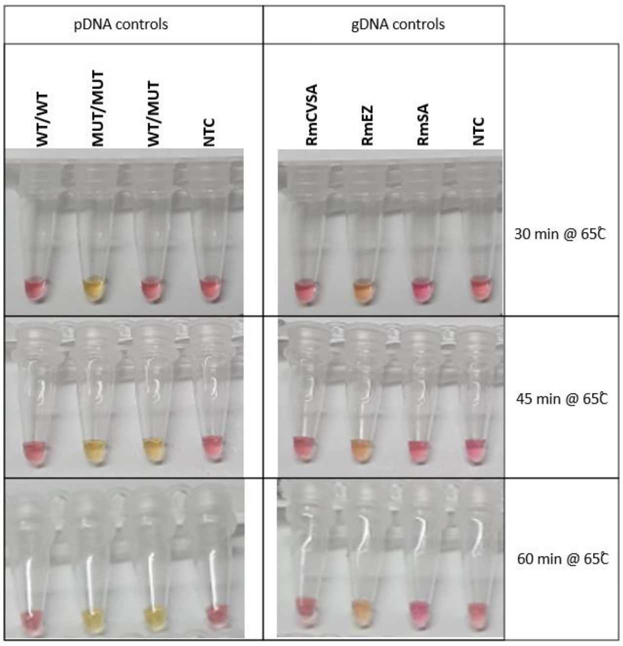

Figure S3. Colorimetric LAMP assessment of the final *R. microplus kdr* assay developed for detection of the SNP in the *R. microplus*-derived genomic DNA. The assays were conducted using linearized plasmid DNA (representing the WT/WT, MUT/MUT and WT/MUT alleles) and genomic DNA (RmCVSA, WT/WT; RmEZ, MUT/MUT; RmSA WT/WT).

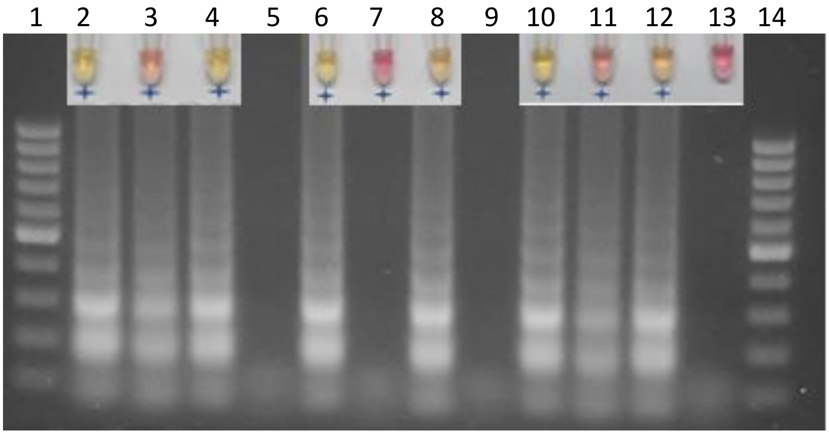

Figure S4. Agarose gel electrophoresis of the *R. microplus kdr* LAMP assay LOD presenting false negative colorimetric results (lanes 3, 11) when compared to actual product formation. Template dilutions represent 5818 copies (lanes 2, 10), 582 copies (lanes 3, 6, 11), 58 (lanes 4, 7, 12) and 6 copies (lane 8). The GeneRuler 100 bp DNA Ladder (lanes 1 and 14) was used to perform size estimation. Images were cropped to include the bottom of each tube containing the reaction mixtures overlaying the corresponding PCR product as analyzed using agarose gel electrophoresis.

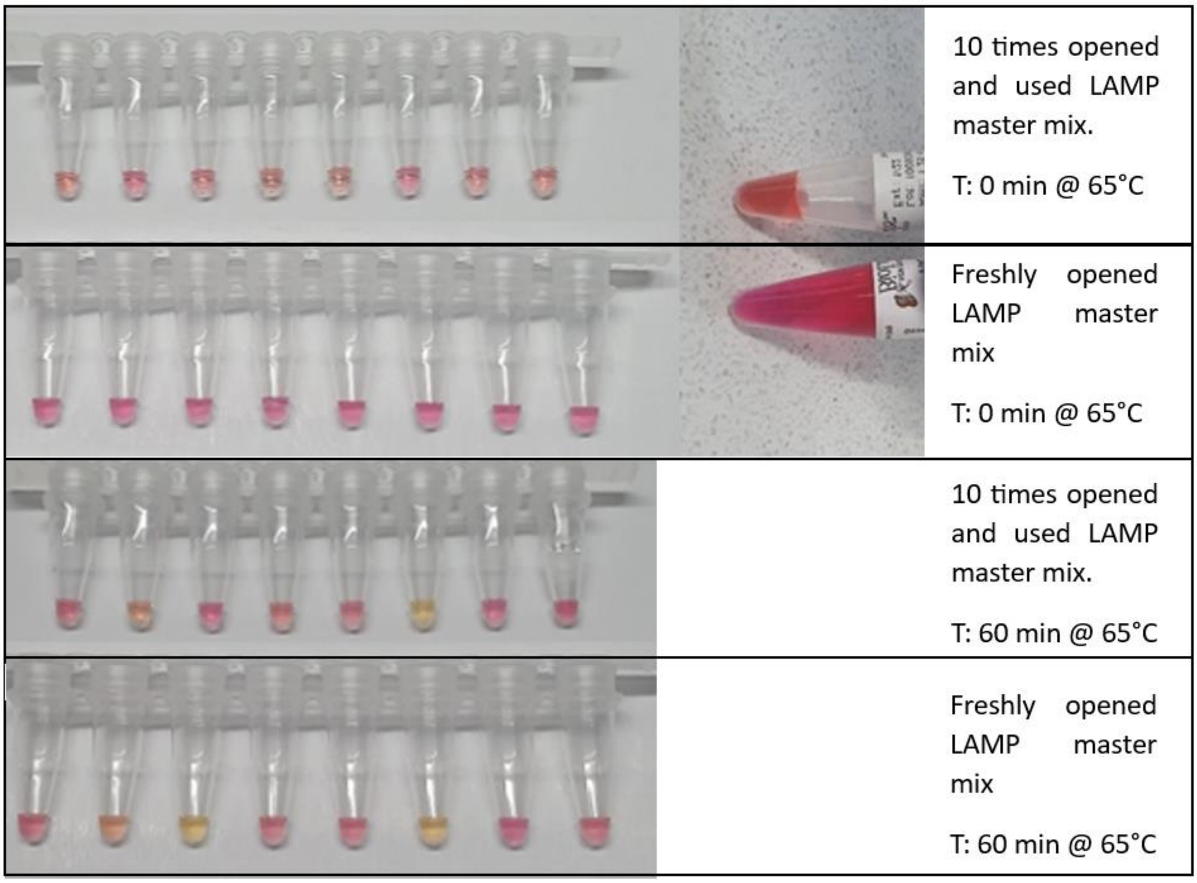

Figure S5. Comparison of frequently opened and freshly opened NEB WarmStart® Colorimetric LAMP 2X Master Mix before and after incubation at 65°C.

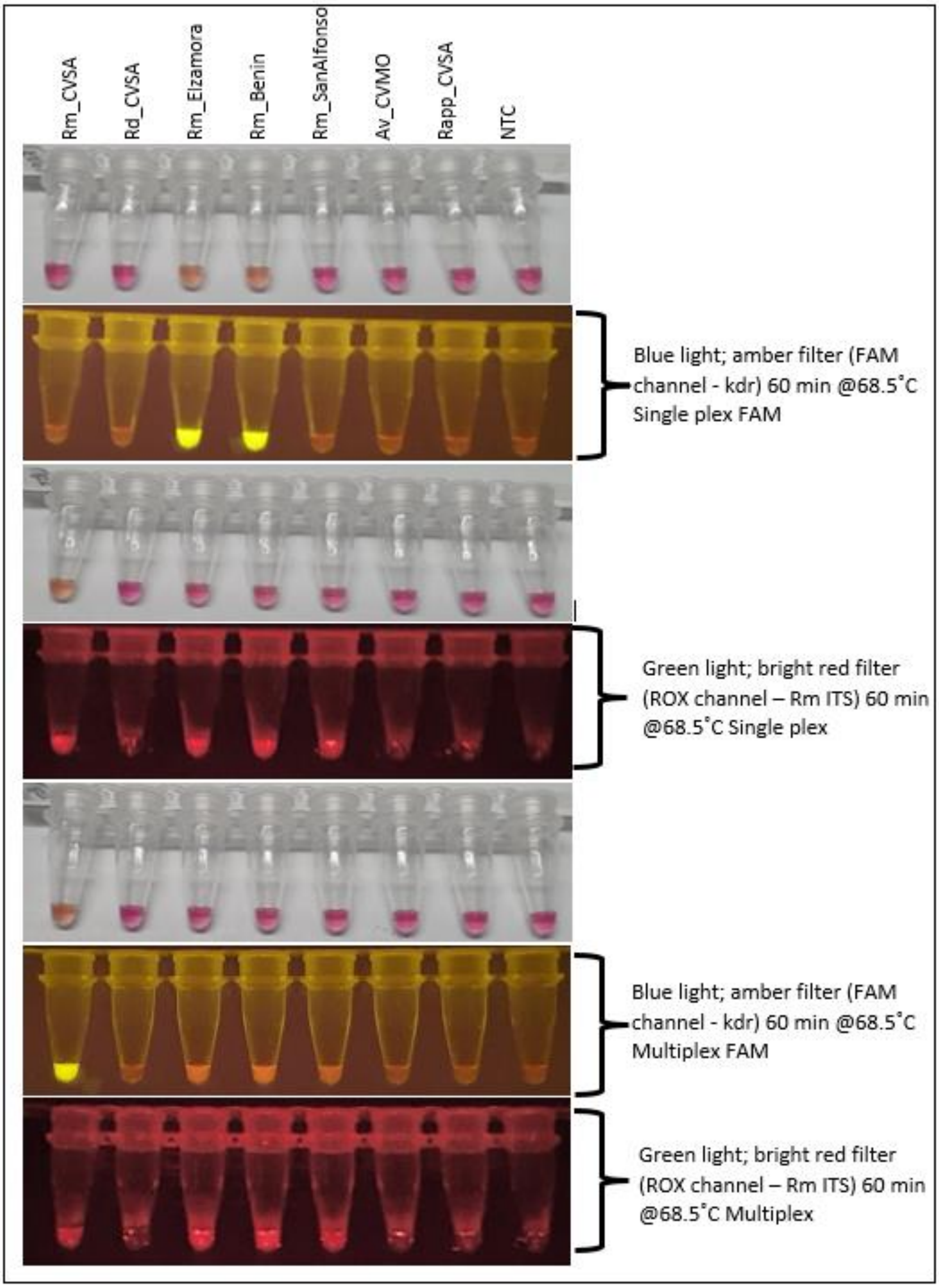

Figure S6. Colorimetric and QUASR detection of the *R. microplus kdr* and ITS2 target regions using LAMP assays as a single plex and multiplex. Rm = *R. microplus*; Rd = *R. decoloratus*; Av = *Amblyomma variegatum*; Rapp = *R. appendiculatus*.

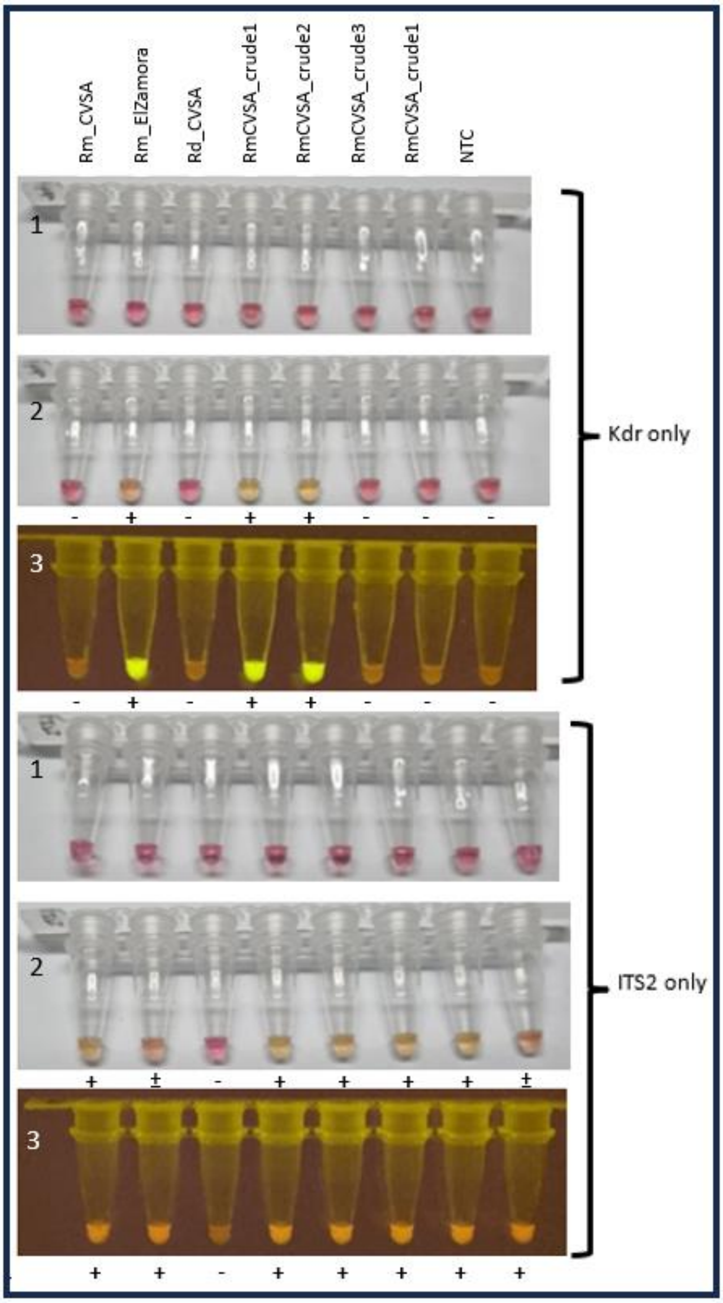

Figure S7. Colorimetric and QUASR detection of the *R. microplus kdr* and ITS2 target regions with purified and crude extracts as templates during the single-target LAMP reactions. The first picture of each set represents the reaction setup prior to incubation, with the second and third pictures representing the colorimetric and fluorescence-based reactions post incubation. Each completed reaction was subjected to visual signal interpretation as indicated with + (present), - (absent) and ± (unsure).

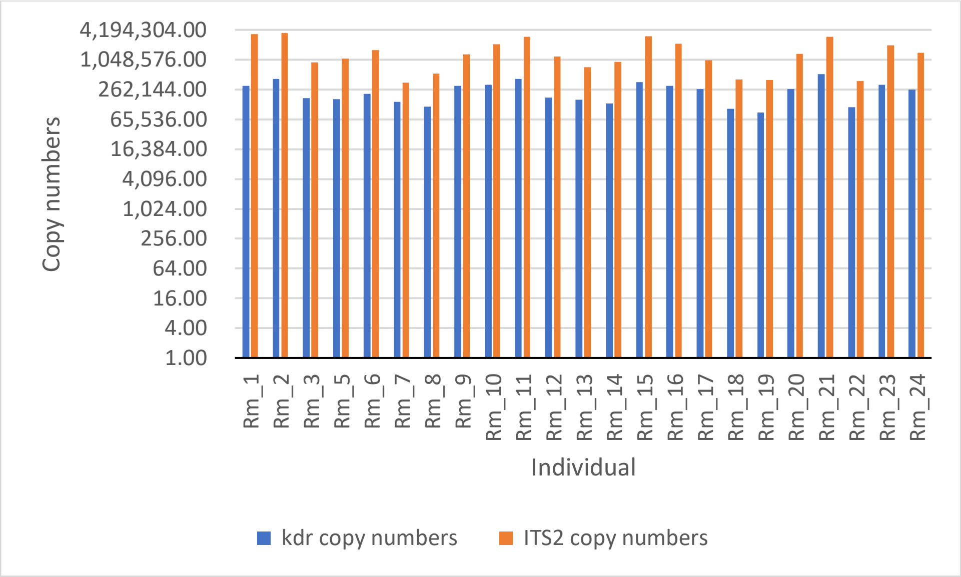

Figure S8. qPCR copy number determination of *kdr* and ITS2 target regions (copies/µl) in a crude extract from individual larvae using a laboratory homogenizer and beads.

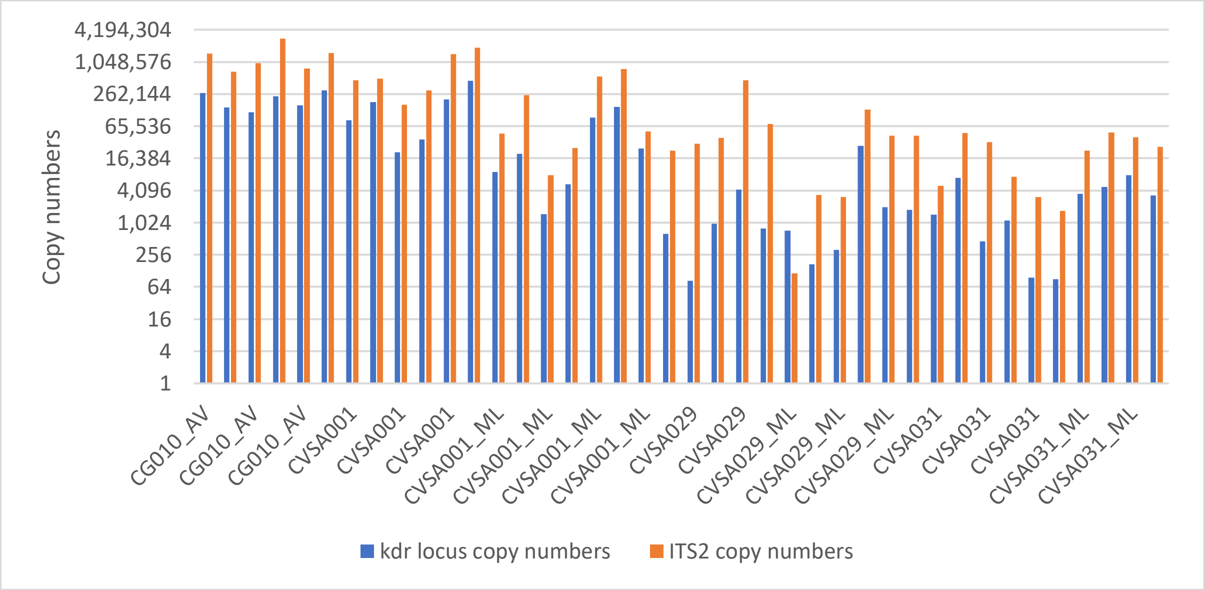

Figure S9. qPCR copy number determination of *kdr* and ITS2 target regions (copies/µl) present in crude extract generated from individual larvae using the field-based homogenization method. Samples indicated with "_ML" represent collaborator samples extracted by an independent operator at a later stage.

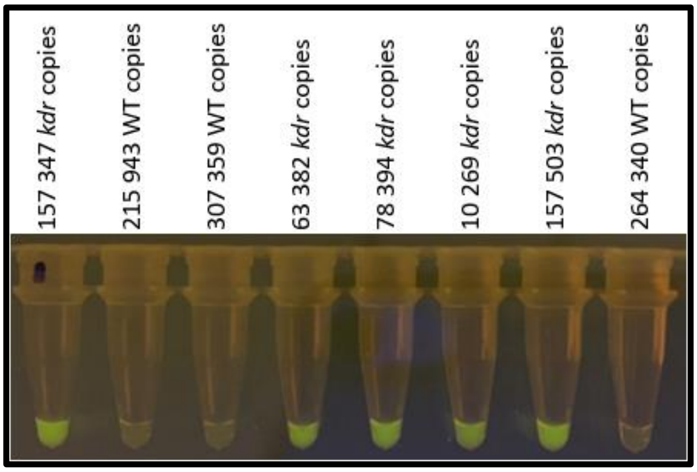

Figure S10. *R. microplus kdr* allele-specific QUASR PCR fluorescent detection of end point PCR product. qPCR *kdr* locus copy numbers [representing the target load in 1 µl crude extract template from individual *R. microplus* larvae (isolate D22-05009)] are indicated above each tube.

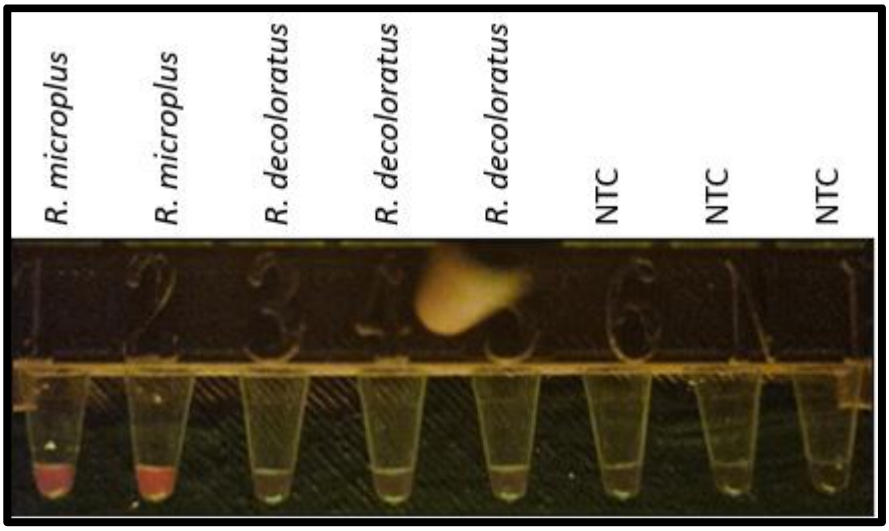

Figure S11. *R. microplus* ITS2-specific QUASR PCR of 1 µl crude DNA extract from individual *R. microplus* and *R. decoloratus* larvae using QUASR fluorescent detection of endpoint PCR products.

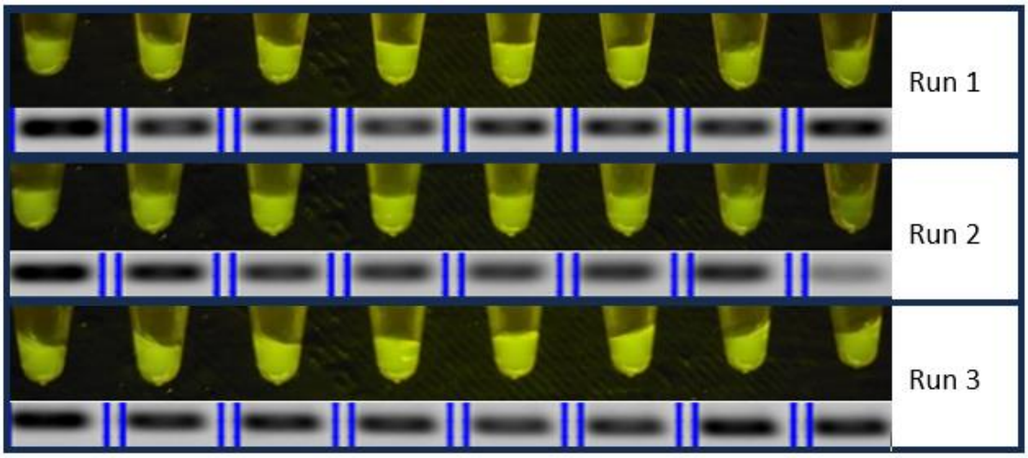

Figure S12. End-point PCR fluorescence and agarose gel electrophoresis analysis of the PCR product. A total of 5 µl of each of the fluorescent reaction mixtures from each tube (position 1 to 8) was subjected to agarose gel electrophoresis (gel image directly below the fluorescent tube). Images were cropped to include the bottom of each tube containing the reaction mixtures and the corresponding PCR product as analyzed using agarose gel electrophoresis.

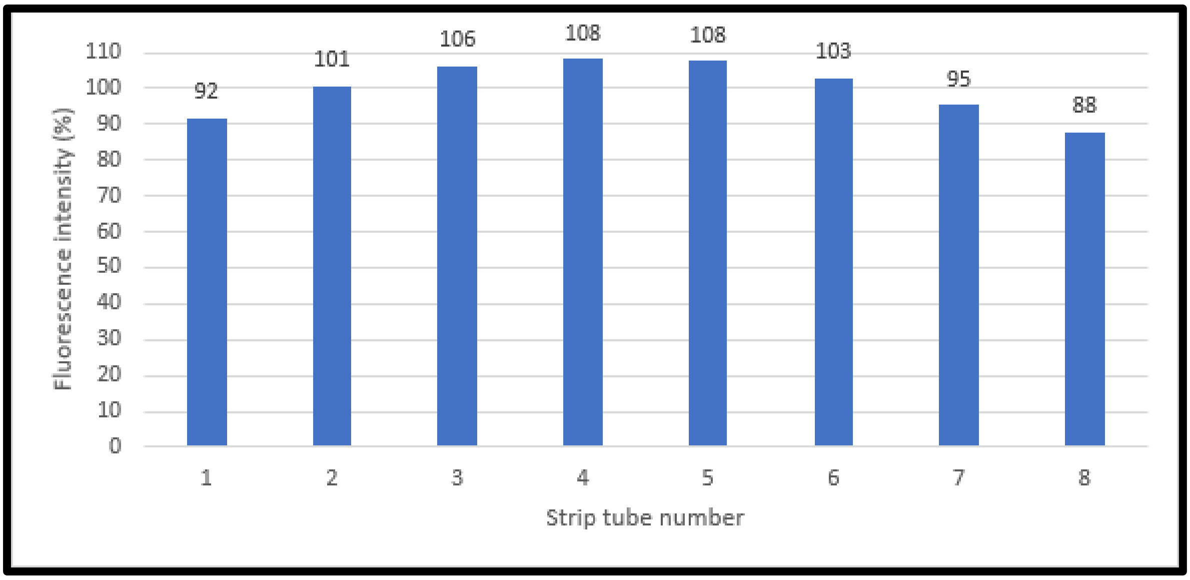

Figure S13. Fluorescence intensity for each tube of the PCR tube strip expressed as a percentage of the average fluorescence intensity for all the tubes calculated using ImageJ.

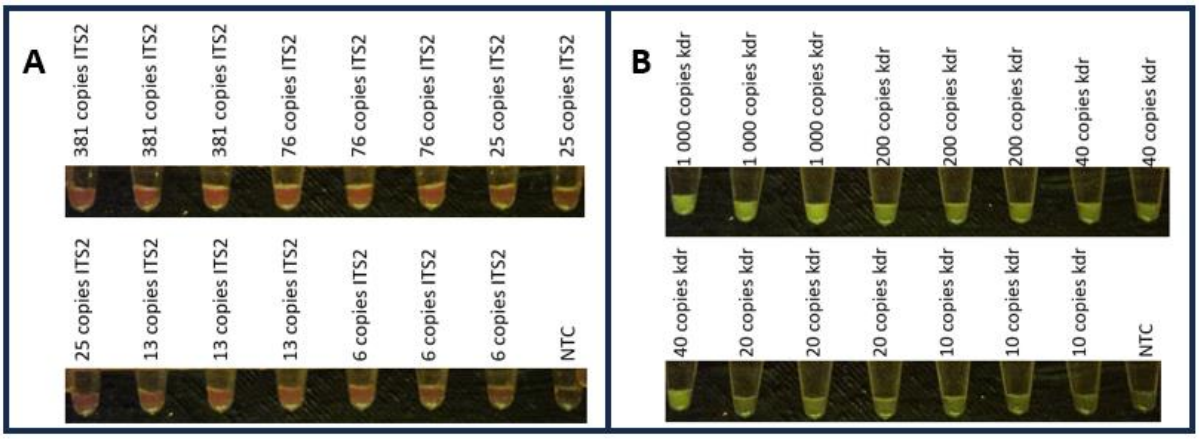

Figure S14. LOD determination of the field-based ARDA using the mini16 thermal cycler and the field-based detection device to detect *R. microplus* ITS2 (A) and *kdr* mutation (B) targets. Images were cropped to include the bottom of each tube containing the reaction mixtures.

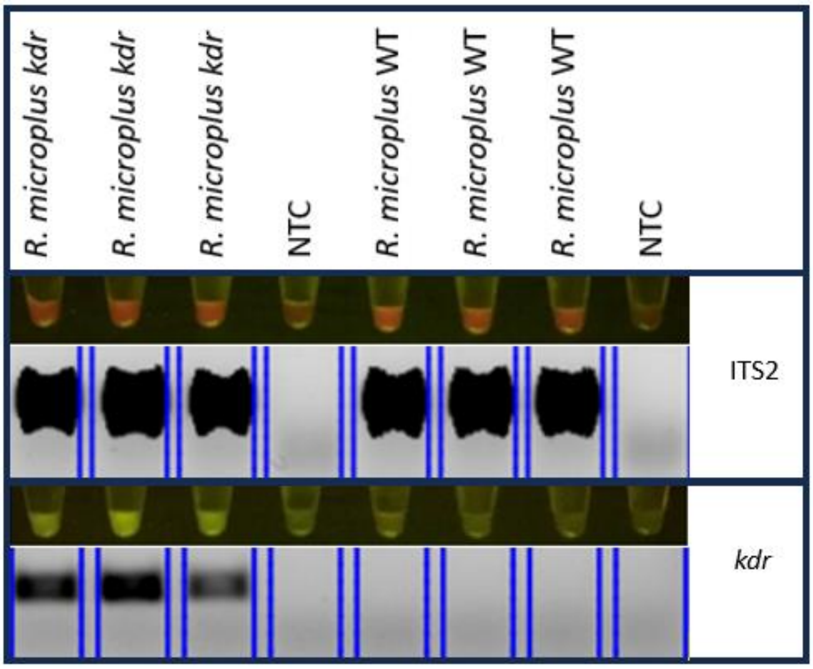

Figure S15. Amplification of the ARDA targets from individual tick larvae using the thawed frozen lysis solution and QUASR PCR premixes. Images were cropped to include the bottom of each tube containing the reaction mixtures and the corresponding PCR product as analyzed using agarose gel electrophoresis.

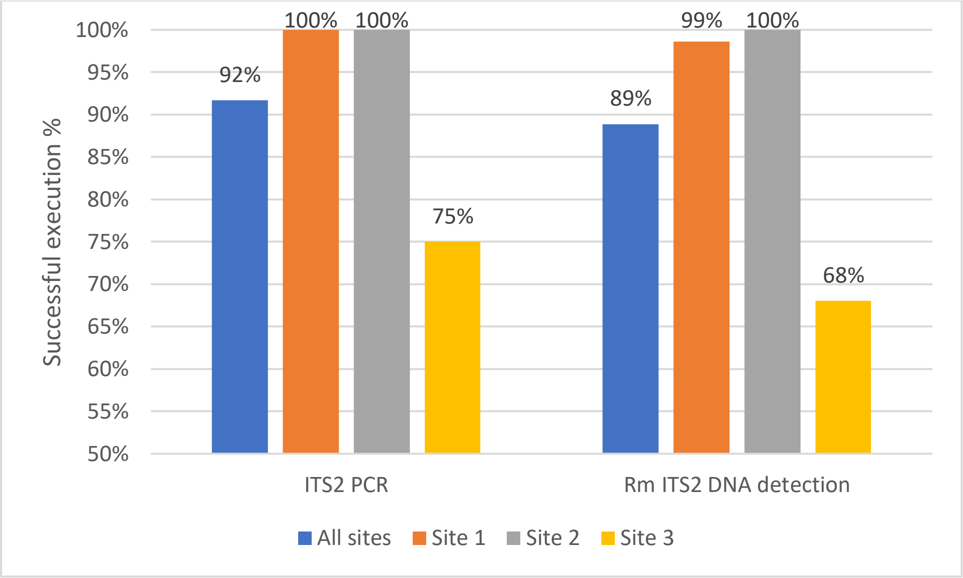

Figure S16. Field-based ARDA execution success as measured by the ability to generate reliable control data as well as the ability to extract amplifiable *R. microplus* DNA.
